# Mycorrhiza-Induced Alterations in Metabolome of *Medicago lupulina* Leaves during Symbiosis Development

**DOI:** 10.3390/plants10112506

**Published:** 2021-11-18

**Authors:** Andrey P. Yurkov, Roman K. Puzanskiy, Galina S. Avdeeva, Lidija M. Jacobi, Anastasia O. Gorbunova, Alexey A. Kryukov, Andrei P. Kozhemyakov, Yuri V. Laktionov, Yuri V. Kosulnikov, Daria A. Romanyuk, Vladislav V. Yemelyanov, Alexey L. Shavarda, Anastasia A. Kirpichnikova, Galina N. Smolikova, Maria F. Shishova

**Affiliations:** 1 Laboratory of Ecology of Symbiotic and Associative Rhizobacteria, All-Russia Research Institute for Agricultural Microbiology, Pushkin, 196608 St. Petersburg, Russia; lidija-jacobi@yandex.ru (L.M.J.); gorbunova.anastasia93@mail.ru (A.O.G.); rainniar13@gmail.com (A.A.K.); kojemyakov@rambler.ru (A.P.K.); laktionov@list.ru (Y.V.L.); kullavayn@gmail.com (Y.V.K.); daria-rom@yandex.ru (D.A.R.); 2 Laboratory of Analytical Phytochemistry, Komarov Botanical Institute of the Russian Academy of Sciences, 197376 St. Petersburg, Russia; puzansky@yandex.ru (R.K.P.); stachyopsis@gmail.com (A.L.S.); 3 Faculty of Biology, St. Petersburg State University, 199034 St. Petersburg, Russia; ekskursbio@gmail.com (G.S.A.); bootika@mail.ru (V.V.Y.); nastin1972@mail.ru (A.A.K.); galina.smolikova@gmail.com (G.N.S.); 4 Center for Molecular and Cell Technologies, St. Petersburg State University, 19934 St. Petersburg, Russia

**Keywords:** *Medicago lupulina*, arbuscular mycorrhiza, *Rhizophagus irregularis*, symbiotic efficiency, plant development, physiological stage, leaf, metabolic profile

## Abstract

The present study is aimed at disclosing metabolic profile alterations in the leaves of the *Medicago lupulina* MlS-1 line that result from high-efficiency arbuscular mycorrhiza (AM) symbiosis formed with *Rhizophagus irregularis* under condition of a low phosphorus level in the substrate. A highly effective AM symbiosis was established in the period from the stooling to the shoot branching initiation stage (the efficiency in stem height exceeded 200%). Mycorrhization led to a more intensive accumulation of phosphates (glycerophosphoglycerol and inorganic phosphate) in *M. lupulina* leaves. Metabolic spectra were detected with GS-MS analysis. The application of complex mathematical analyses made it possible to identify the clustering of various groups of 320 metabolites and thus demonstrate the central importance of the carbohydrate and carboxylate-amino acid clusters. The results obtained indicate a delay in the metabolic development of mycorrhized plants. Thus, AM not only accelerates the transition between plant developmental stages but delays biochemical “maturation” mainly in the form of a lag of sugar accumulation in comparison with non-mycorrhized plants. Several methods of statistical modeling proved that, at least with respect to determining the metabolic status of host-plant leaves, stages of phenological development have priority over calendar age.

## 1. Introduction

Arbuscular mycorrhiza (AM) is one of the oldest forms of widespread symbiosis and was a factor in the land invasion of plants more than 400 million years ago [1]. Most land plants form this symbiosis with fungi from the Glomeromycotina subdivision in the Mucoromycota division [2]. AM promotes plant growth by enhancing mineral (primarily phosphorus) nutrition [3] and increases plant adaptive fitness to biotic and abiotic factors [4,5,6,7]. AM fungi are obligate symbionts and receive 4–20% of their photosynthetic products from the host plant in the form of glucose [8,9] and sucrose [8,10]. The intensity of the host plant’s phosphate and carbohydrate metabolism in AM defines whether the interaction between symbiotic partners is mutualistic or parasitic [3,11,12]. Recent investigations have revealed the possible signaling function of sugars and Pi starvation in the establishment of AM [8,13,14]. Close interaction between sugar and hormone signaling extend the possible means by which plant growth and development are regulated as well modulating the establishment of AM [15].

The complexity of this above organismic system requires a new integral method of analysis. Recently, plant-AM fungi interactions have been subjected to a new systemic approach. The full-scale transcriptome of *M. truncatula* [16,17] elaborated its alterations during AM formation and subsequent development [18,19,20]. Particular interest was paid to further functional protein analysis. A number of studies revealed several symbiosis-specific protein groups: the AM-specific phosphate transporter MtPT4 in *M. truncatula* [21,22], MtPT8 [23], AM-specific ATPase, Mtha1 [19,24], Mtsucs1 sucrose synthase, and sucrose biosynthesis enzymes [25]. Sometime later, full metabolic profiling was performed on *M. truncatula* roots. This revealed fatty acids that were particular to AM [26]. AM plants were characterized by an increase in the total content of carbohydrates, glucose, and starch [27].

Later on, the effect of *R. irregularis* inoculation on the metabolic profile of *Senecio jacobaea* shoots and roots at the 70th DAI (10th week after treatment) was examined [28]. A total of 33 compounds (including seven apocarotenoids) were shown to be accumulated in the roots of colonized plants. However, the absence of significant changes in metabolites was found in all OPLS-DA models of ragweed shoot tissues due to AM colonization [28]. A larger set of root metabolites (>600) was studied at the 50th DAI (day post inoculation) in *Solanum lycopersicum* L. cv. Castlemart [29]. Significant changes in metabolite profiles were found in the roots of the *spr2* mutant with modified levels of *R. irregularis* mycorrhization [29]. *M. truncatula* cv. Jemalong J5 plants inoculated with *R. irregularis* (strain BEG141) showed an increase in the biosynthesis of flavonoids, terpenoids, jasmonic and abscisic acid with a decrease in the biosynthesis of salicylic acid, as well as an increase in the expression of transcription factor MYC2, the main regulator of JA-dependent reactions [30]. Data were obtained at 28 DAI (4 weeks post-inoculation). 

Thus, to summarize, the plant metabolome varies distinctly depending on the conditions of mycorrhization and plant species. Significant AM-induced changes in the root metabolome are not always accompanied by changes in the shoot metabolome [28]. Unfortunately, studies of the AM effect on the plant metabolome were non-systemic and did not test for stages of host plant development. Rather, samples were taken at randomly selected days after inoculation or sowing [29,30,31,32,33]. The data obtained for some plant species is therefore difficult or sometimes even impossible to apply to other species [27]. For this reason, the general mechanisms controlling the metabolism of different plant species in AM have yet to be identified. 

The collected data do not always allow for the determination of whether alterations in metabolic profiles were caused by AM establishment or host-plant development. Recently, the effect of mycorrhization with *R. irregularis* on metabolic and physiological alterations was analyzed in the leaves of peas (*P. sativum*) at six key stages of host-plant development from the 7th to 110th DAI [34,35]. It was significant even though this cv. Finale formed an ineffective symbiosis with *R. irregularis* [34]. It was discovered that AM partially suspends senescence of the leaves (i.e., prolongs the period of “youth” of the leaves) and possibly leads to better accumulation of metabolites such as amino acids and unsaturated fatty acids. 

How this metabolic AM effect would be modified in the case of effective AM symbiosis is very questionable. The study is aimed at defining alterations in the leaf metabolome of the highly mycotrophic *M. lupulina* MlS-1 line from the early to late stages of plant development under conditions of low phosphorus levels in the substrate.

## 2. Results

### 2.1. The Effect of Symbiosis with R. irregularis on Phosphate Accumulation in the Ecologically Obligate Mycotrophic M. lupulina MlS-1 Line under Low Pi Conditions

**Figure 1:**
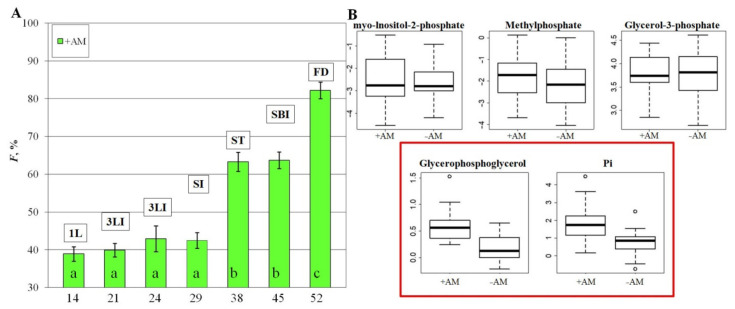
AM frequency in *M. lupulina* roots (*F)*, (**A**) at different development stages, phosphate levels in *M. lupulina* leaves (**B**) at common development stages in “−AM” (without AM) and “+AM” (inoculated with *R. irregularis*). “1L”, “3LI”, “SI”, “ST”, “SBI”, “FI”, “FD”—see Table 1 in Section 4; Different letters (a–c) indicate significant differences within the same mycorrhizal parameter (ANOVA and Tukey’s test; *p* < 0.05). The average values (log_2_ of means of tree replicates) with standard errors are presented. The axis of the abscissa shows the days after sowing (DAS).

The study showed that in inoculated *M. lupulina* plants, the frequency of AM fungus *R. irregularis* mycorrhizal infection (*F*) continuously increased throughout the entire life cycle of *M. lupulina* (Figure 1A), a significant (*p* < 0.05) *F* value increase was observed at the stooling (ST) stage, by 1.5 times, and in the flowering development (FD) stage, by 1.3 times. The data correspond to the previously published detailed development of AM structures for this model object (intraradical mycelium, arbuscules, and vesicles of AM fungus) [12].

As can be seen from Table 1, the development of AM plants was ahead of control plants without AM by one stage already starting from the second stage up to flowering. Mycorrhization led to a more intensive accumulation of phosphates in the leaves of *M. lupulina* with AM vs. non-inoculated control plants. The analysis showed that not only plants of the same stage (same DAS) but also plants of the same development stage with and without AM had significant differences in the content of two out of five tested phosphates—glycerophosphoglycerol and Pi (Figure 1B). The content of glycerophosphoglycerol was 5.6 times higher, and Pi was 2.0 times higher in plants with AM. The reliability of the differences was confirmed by the *p*-value (*p* = 0.002 and 0.003, respectively) and false discovery rate (FDR = 0.007 for both) in Mann–Whitney–Wilcoxon analyses. VIP > 1 in OPLS-DA assays indicates a significant effect of AM on Pi and glycerophosphate accumulation in plants. Hence, *R. irregularis* inoculation stimulated the accumulation of these forms of phosphates not only in total for all general stages of development but also for each stage separately (1L, SI and FI), and the response to mycorrhization was observed early, already at the first detection stage, 1L at 14 DAS.

**Figure 2:**
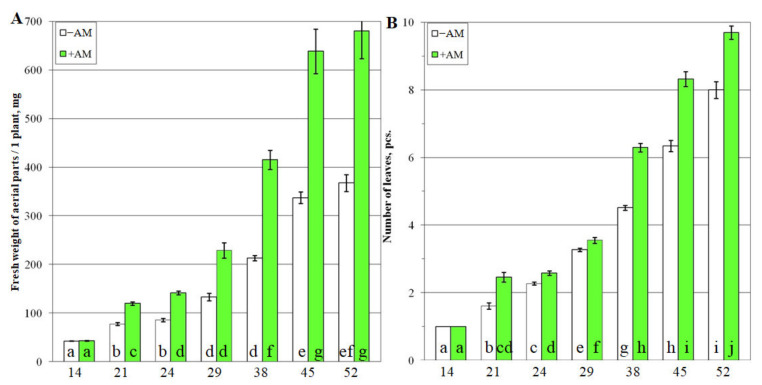
Fresh weight of aerial parts (**A**) and number of leaves (**B**) per one *M. lupulina* plant. “−AM” is the variant without AM fungus inoculation, “+AM” is the variant inoculated with *R. irregularis* AM fungus. Different letters (a–j) indicate significant differences within the same parameter of the productivity (ANOVA and Tukey’s test; *p* < 0.05). The axis of the abscissa is DAS, see other notes in Figure 1.

Increased phosphate uptake in AM plants compared to control (“−AM”) was one reason for the significant increase in *M. lupulina*’s productivity parameters, such as the fresh weight of aerial parts and the number of leaves (Figure 2), as well as the height of the main stem and the number of internodes on the main stem (Figure S1). A significant (*p* < 0.05) early response to mycorrhization was observed for the fresh weight of aerial parts and the number of leaves on the main stem already from the second stage (21 DAS; Figure 2A,B); the symbiotic efficiency of these parameters was high (>50%) and significant at 21 DAS (third leaf initiation stage, 3LI), followed by a maximum efficiency at 38 DAS (shoot branching initiation stage, SBI) (Figure S2). A significant (*p* < 0.05) response of the height of the main stem and the number of internodes on the main stem was observed from the very beginning of their development (from 24 DAS; Figure S1A,B); the symbiotic efficiency of these parameters had one maximum at 38 DAS, especially the plant stem height (+203.4%) (Figure S2). Thus, the symbiotic efficiency of the formed AM was already high from the earliest stages, and MlS-1 line plants should be considered ecologically obligatory mycotrophic under conditions of a low level of Pi in the substrate [12,36,37].

### 2.2. Effect of R. Irregularis on the Photochemical Activity, Pigment Content and Leaf Area of the M. lupulina MlS-1 Line

**Figure 3:**
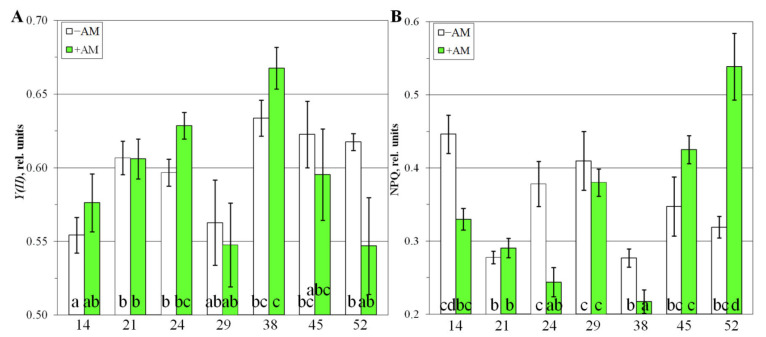
The maximum photochemical quantum yield of photosystem II, observed after dark acclimation (*Fv*/*Fm*); (**A**), non-photochemical quenching in *M. lupulina* leaf tissues (*NPQ*); (**B**). Different letters (a–d) indicate significant differences within the same parameter of the photosynthesis (ANOVA and Tukey’s test; *p* < 0.05). The axis of the abscissa is DAS, see other notes in Figure 1.

The analyzed plant-microbial system (PMS) “*M. lupulina* + *R. irregularis*” is highly effective both for the host plant and for the AM fungus, which continues to actively spread in the root system (Figure 1A) until the final stage of analysis, i.e., flowering development (FD) and “green pod initiation” stage. It should be assumed that the products of photosynthesis in such PMS are in demand by both symbiotic partners. Therefore, the analysis of photochemical activity and the content of pigments in the youngest fully formed leaf was carried out, as well as the assessment of the leaf area of *M. lupulina* (Figure 3 and Figure S3).

The maximum quantum yield of the PSII (*Fv*/*Fm*; Figure S3A) was significantly (*p* < 0.05) higher in the leaves of AM plants compared with the plants without AM at 24–38 DAS (Figure S3A). This indicates a higher potential photosynthetic activity in *M. lupulina* at the ST stage in AM plants. The effective quantum yield of PSII (*Y(II)*) characterizes the real photochemical activity of tissues. The effective quantum yield of PSII (*Y(II)*) was lower than *Fv*/*Fm* (~0.55–0.65; Figure 3A) and did not differ in control and inoculated plants at 14–21 DAS. However, it was higher in inoculated plants at 24 DAS and 38 DAS, and lower at 45 DAS and 52 DAS. The dramatic drop in this value at 29 DAS is notable.

The estimation of quenching non-photochemical fluorescence relied on the coefficient *NPQ*, which is essential for protecting the machinery of photosynthesis from possible damage by intense light. *NPQ* was ~0.2–0.5 at different stages of plant development. *NPQ* was characterized by lower values for AM plants at 14, 24, 29, and 38 DAS. Therefore, in inoculated plants, the light energy was used more for photochemical reactions than for non-photochemical ones. At the final stages of development (FI and FD; 45 and 52 DAS), this was significantly increased in AM plants (Figure 3B), which may be due to the processes of faster aging of plant leaves during mycorrhization. At the same time, the leaf area of AM plants during the development of lateral branches (SBI) at 29–38 DAS had an intensive increase of 2.3 times (Figure S3B). The AM symbiotic efficiency (calculated by the increase in leaf area) during the initiation of stooling (SI) from 24 to 38 DAS had the greatest increase by 2.9 times from +57.5% to +164.7% (Figure S2).

It should be noted that the relationship of the indices of photochemical activity is interconnected with the content of chlorophylls. Only in the period from 24 to 38 DAS was there a significant excess of chlorophyll a and b content in the AM plant leaf tissues vs. control (−AM) (Figure S4). The highest content of pigments, including the amount of chlorophylls and the amount of carotenoids, was observed at 38 DAS in plants with AM at the SBI stage (Figure S4). At the same time, the ratio of the sum of carotenoids to the sum of chlorophylls was higher in plants without AM in comparison with AM plants at 14 and 24–38 DAS (Figure S5).

The data presented—including *M. lupulina* root mycorrhization levels, leaf phosphorus content, productivity parameters, photosynthesis activity, and pigment content—allow for the assumption that one of the most important development stages of efficient AM symbiosis is the transfer of plants into the stooling stage (ST): 24 DAS (SI), 29 DAS (ST), and 38 DAS (SBI). At 38 DAS (SBI), a highly efficient AM symbiosis was formed between *M. lupulina* and *R. irregularis*.

### 2.3. General Characteristics of Metabolite Profiles of M. lupulina Leaves

The metabolite profiles of *M. lupulina* leaves included 320 metabolites, of which 83 were identified exactly and only the class of compounds was determined for another 80 (Tables S1 and S2). Sugars (86 metabolites), including pentoses, hexoses, and oligosaccharides and their derivatives, such as sugar alcohols and sugar acids, and other sugar derivatives were most widely represented in the determined profiles. 25 amino acids (including all proteinogenic), 12 carboxylic acids, energy metabolism intermediates, 8 fatty acids and their derivatives, as well as nitrogenous bases, sterols, etc., were also identified.

One of the key tasks was to assess changes in the general *M. lupulina* metabolic profile with reference to the plant development stage and the resulting influence of mycorrhization. First of all, metabolite profiles were represented in low dimension space to unveil the similarities between them. Two methods based on different mathematical principles were used. First, PCA, as the most common method in metabolomics, was applied and based on the representation of metabolite content dispersion. Secondly, MDS (multidimensional scaling) was used. This method is less common and based on maintaining pairwise distances between observations. The use of metabolite content for comparative description of metabolomes is not always entirely good as it depends on normalization. This limitation can be overcome via the correlation coefficient as a measure of similarity. Therefore, we augmented our analysis by using MDS with Pearson’s distance (1–r).

**Figure 4:**
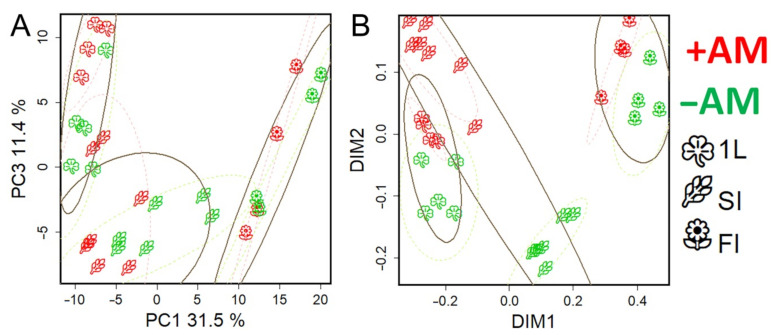
Representation in low-dimension spaces of metabolite profiles revealed from leaves sampled −AM and +AM plants at comparable stages. (**A**) PCA—score plots, %—percent of variance. (**B**) MDS—metabolite profiles in the space revealed using multidimensional scaling (MDS) with 1–*r* as a measure of distance between observations, where *r* is Pearson’s correlation coefficient. Ellipses—90% CI.

It can be seen that the profiles were grouped according to the development stage. This trend was more clearly observed when the dimensionality was reduced using MDS (multidimensional scaling) with the Pearson’s distance (Figure 4A and Figure S6) or the Euclidean distance (Figure 4B and Figure S6). Analysis showed that the development at the metabolic level is a complex nonlinear and uneven process. The nonlinearity of the development is expressed in the fact that the direction of changes varies, sometimes even to orthogonal, which indicates deep differences in metabolic changes between different stages from leaf formation to the stages of flowering and fruiting. However, the profiles showed some tendency to shift in time along PC1, explaining 33% of the variance. Thus, we can suppose some general trends in development. Note that the 1L and 3LI stages were quite similar, whereas stooling was accompanied by significant changes. In the case of +AM plants, the difference between the 1L and SI stages looked less significant than in the control plants (−AM). Large-scale metabolic changes that occurred in control plants (−AM) during the transition from 3LI to SI were postponed in +AM plants for the period from SI to ST. The branching stage turned out to be quite similar to stooling, while the transition to flowering and fruiting was again accompanied by significant rearrangements.

It is important to note that the metabolic profiles in the +AM variant compared to −AM at the same stage shifted towards physiologically younger plants. This effect was especially pronounced at the SI stage.

### 2.4. Analysis of Changes in Leaves Metabolite Profiles during Mycorrhized and Control M. lupulina Plants during Development

#### 2.4.1. Changes in the Metabolite Profiles of *M. lupulina* Leaves

**Figure 5:**
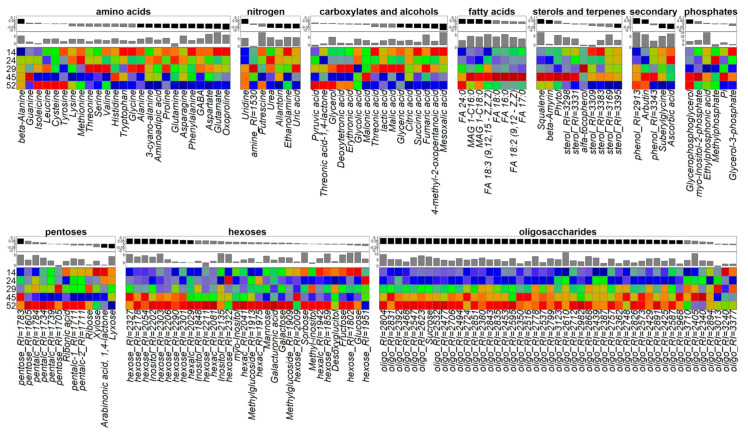
The dynamics of mean metabolites levels in −AM plant leaves of *M. lupulina*. Data were normalized per sample median, log-transformed, and standardized. Inside class metabolites sorted by OPLS (with DAS as response) loadings (*p*), represented at the upper barplot (black bars—loadings with VIP > 1, positive values—increasing with aging). Lower barplot—MDA, mean decrease accuracy from random forest. DAS—day after sowing. Contractions: MAG—monoacylglycerol, FA—fatty acid, oligo-complex carbohydrates (di-, oligosachrides, molecules with sugar parts), ni—not identified, RI—retention index.

The identification of general patterns of metabolite content dynamics was provided through OPLS modeling using the age of plants as responses. The parameters of −AM and +AM plant models are very similar. In both cases, about 30% of the variance was associated with the predictive component. Figure 5 shows a heat map of the normalized metabolite content in −AM control plants. The barplots of the OPLS model factor loadings of the predictive component and mean decrease accuracy from random forest are located on top. Most of the identified metabolites showed positive loadings, which was a consequence of their accumulation during development. This effect was achieved mainly due to sugars, mostly oligosaccharides, which were accumulated at later stages. In contrast, a decrease was observed in the content of carboxylic acids, amino acids, and other nitrogen-containing compounds.

In order to compare the changes in the +AM and −AM plants metabolite profiles, a SUS-plot (shared and unique structures) graph was constructed wherein the metabolites are represented in the factor loading space of the two OPLS models described above (Figure S7). It can be seen that a small number of metabolites exhibited noticeable differences in the value of loadings. Thus, during mycorrhization, there was no consistent accumulation of fatty acids and phosphate over time. In addition, mycorrhization did not reduce the content of certain amino acids (GABA, tryptophan) and carboxylates (fumarate, citrate, succinate, and malate). However, it is obvious that most of the metabolites had the same signs of loadings and there is a correlation between them (Spearman’s correlation coefficient *ρ* = 0.77, *p* = 10^−15^). The correlation and the same signs indicated the similarity of the changes that occur during development.

#### 2.4.2. The Role of the Phenological Stage in the Metabolite Profile Determination

**Figure 6:**
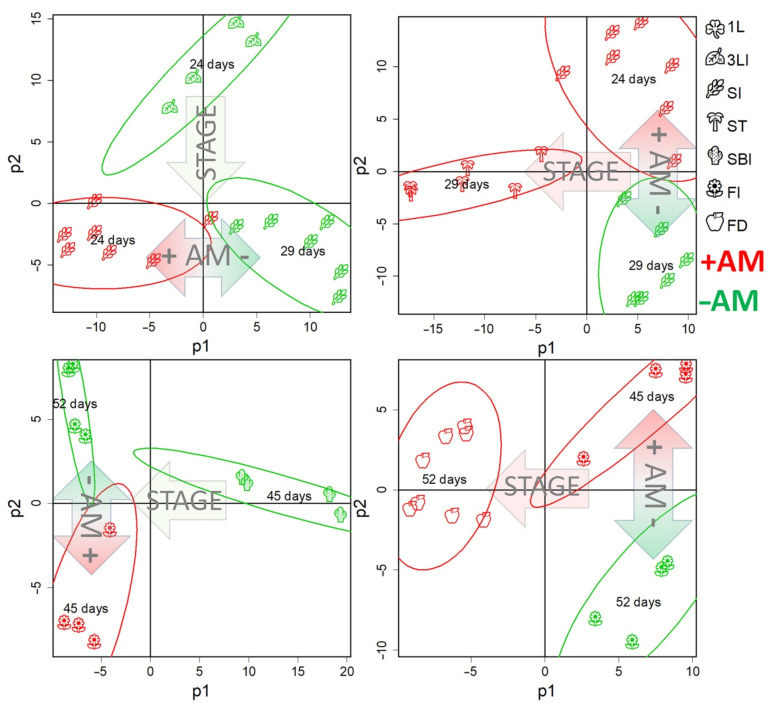
The role of the phenological stage and calendar age (DAS) of plants on metabolite profiles. Figures represent score plots of PLS-DA models of four groups of observations consisting of three classes, two of which had the same calendar age (DAS) and different stages of development and two had the same developmental stage but different ages.

The age of a plant can be determined not only by a calendar day, for example, the day after inoculation or sowing (DAS/DAI), but also by the phenophase onset (the stage of plant development). In addition to its fundamental biological significance, this question is of particular importance in the comparative analysis of plants developing under different conditions, since the answer determines the correctness of the choice of time point pairs for comparing experimental plants. In this study, a PLS-DA analysis was conducted involving four observation groups each consisting of three classes. Two of these groups had equal DAS and different developmental phases, and two had the same developmental phase and different DAS. As can be seen from Figure 6, in which the profiles are scattered in the space of the accounts of the first two predictive components, the action of AM was associated with one of them and development was associated with the other. In both cases, plants that were in the same phase, but of different ages, were closer to each other in the space of the component with which development was associated. Thus, the metabolomic analysis indicates that the metabolic status is determined more by the phenological phase of development than the calendar age. Therefore, it is necessary to choose plants corresponding to one phenophase for a comparative analysis.

#### 2.4.3. The Effect of Mycorrhization on Leaves of the Metabolite Profile at First Leaf Stage

**Figure 7:**
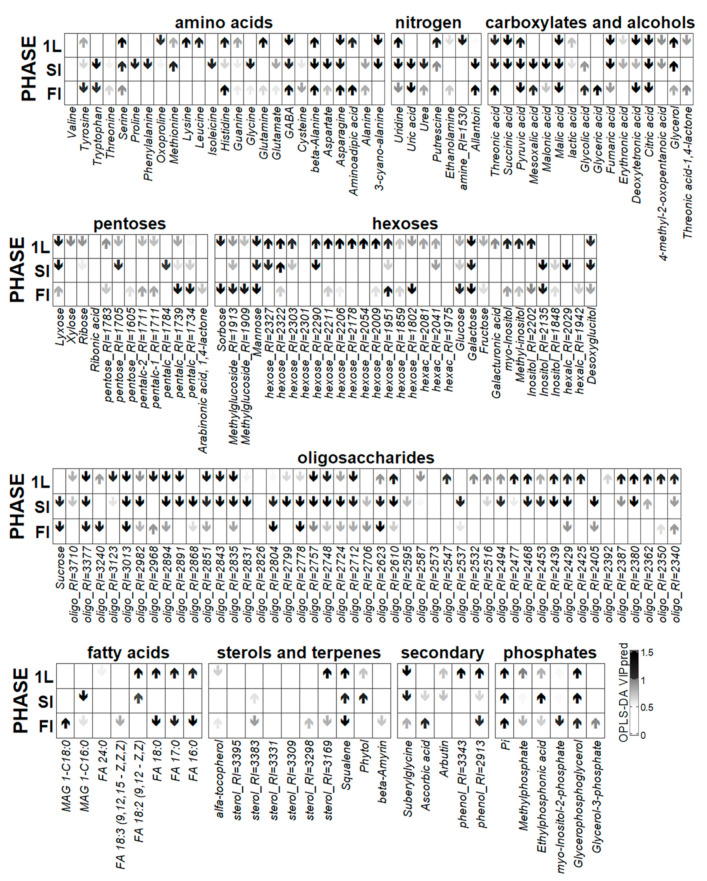
Mycorrhization effects on metabolite profile at stages of first leaf (1L), stooling initiation (SI), and flowering initiation (FI). Heatmap of OPLS-DA loadings (of predictive component), *p*. Up arrows refer to positive *p* corresponding to level increased under mycorrhization.

The constructed OPLS-DA model included two orthogonal components: R^2^X = 0.79, R^2^Y = 0.99 (*p* = 0.03), Q^2^Y = 0.88 (*p* = 0.03). A total of 28% of the variance was associated with the mycorrhization status. Figure 7 shows graphs of the loadings of the predictive component (an up arrow indicates that the loading sign corresponds to a higher level during mycorrhization). At the 1L stage, mycorrhization contributed to the accumulation of certain amino acids: lysine, leucine, serine, glutamine, and some other nitrogen-containing compounds. During mycorrhization, the content of fatty acids increased. In the +AM plant leaves, a higher content of unidentified hexoses was observed and more phosphates accumulated.

The control (−AM) plants were characterized by a higher content of carboxylates, such as fumarate, succinate, and citrate, as well as some amino acids such as GABA, cyanoalanine, oxoproline, and arginine. The distinctive features of the −AM plant profiles included the accumulation of pentoses: ribose, xylose, and lyxose.

In order to identify the biochemical pathways that are most susceptible to AM, a set enrichment analysis [38] was performed using the predictive component loadings from the OPLS-DA model for ranking. As can be seen (Figure S8), mycorrhization leads to an increase in the activity of pathways associated with the metabolism of carbohydrates and sterols. On the other hand, there was a repression of the activity of the tricarboxylic acid cycle (TCA cycle) and pathways associated with the exchange of carboxylates and amino acids.

#### 2.4.4. Metabolomic Differences between the First Leaf (1LI) and the Stooling Initiation (SI) Stages

##### The Changes in the −AM Plant Metabolomes

For nonmycorrhized plants, there were two stages for this period which lasted from 1L to 3LI (14–24 DAS) and from 3LI to SI (24–29 DAS). The designed OPLS-DA models included two orthogonal components for the classification of 1L–3LI: R^2^X = 0.74, R^2^Y = 0.99 (*p* = 0.03), Q^2^Y = 0.89, (*p* = 0.01); 3LI–SI: R^2^X = 0.78, R^2^Y = 0.99 (*p* = 0.02), Q^2^Y = 0.90, (*p* = 0.03). For the first stage, 22% of the variance was associated with the predictive component, and in the case of the second stage, it was −25%. Thus, despite the fact that the second stage was twice as short as the first, the changes were more pronounced. This might indicate large-scale changes in metabolism during the transition to a new stage of morphogenesis. This was consistent with the representation of profiles in a low-dimensional space, wherein stooling initiation observations were removed from those at the previous stage (Figure 4 and Figure S6).

**Figure 8:**
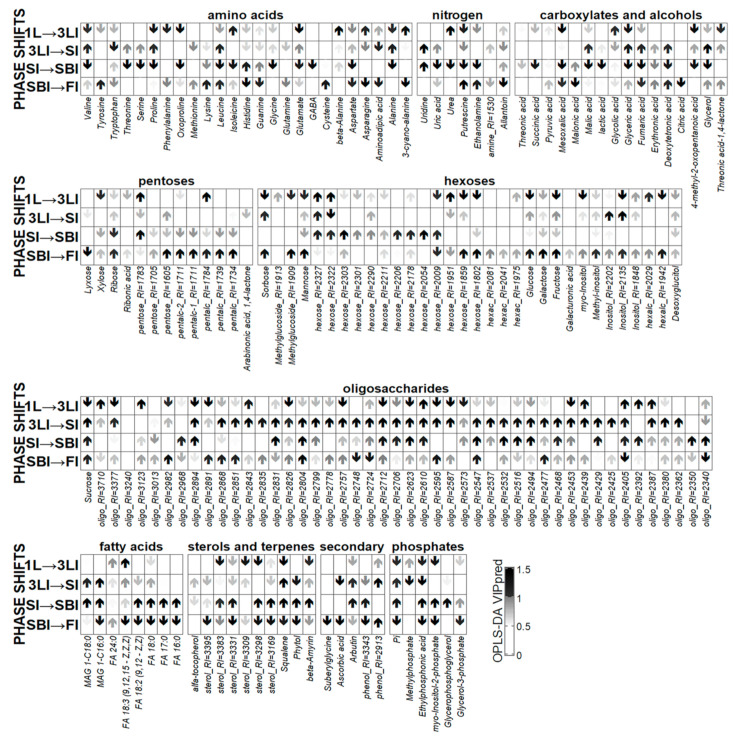
Metabolomic changes accompanying transfer between stages in −AM plants. Heatmap of OPLS-DA loadings (of predictive component), *p*. Up arrows refer to positive *p* corresponding to level increased at the later phase.

As can be seen from Figure 8, quantitative changes in the oligosaccharide content occurred during the formation of leaves; the content of sucrose and major hexoses (fructose and glucose) decreased in particular. Multidirectional trends were revealed for amino acids: the levels of glutamate, oxoproline, phenylalanine, proline, and alanine decreased, and the level of *β*-alanine, asparagine, and cyanoalanine increased. Only a small portion of the carboxylates was characterized by significant changes in content: the level of glycolate increased and the level of mesoxalate and glycerate decreased. There was also a notable decrease in phosphates during this period. Sterols showed a content decrease against the background of an increase in the level of linolenic acid. Enrichment analysis revealed that no pathways were significantly associated with metabolome changes during this period, apparently due to the multidirectional metabolite content changes.

In contrast to the leaf formation stage, when the dynamics of various groups of compounds was complex, and sometimes multidirectional, the process became much more uniform during the transition to stooling. Most differences were associated with the intensive accumulation of oligosaccharides, including sucrose. An increase in the acylglycerol content was observed and the linolenic acid content continued to increase during this stage. Furthermore, enrichment analysis showed that this stage featured an activation of various pathways associated with the starch and hexoses metabolism (Figure S8).

Differences between 1L–3LI and 3LI–SI illustrate the factor loadings contrast (Figure S9), between which there is a weak but negative correlation (*ρ* = −0.28, *p* = 10^−4^). Thus, during this period, there was variation in the dynamics of metabolites, reflecting changes in the development vector.

##### The Changes in the + AM Plants Metabolome

**Figure 9:**
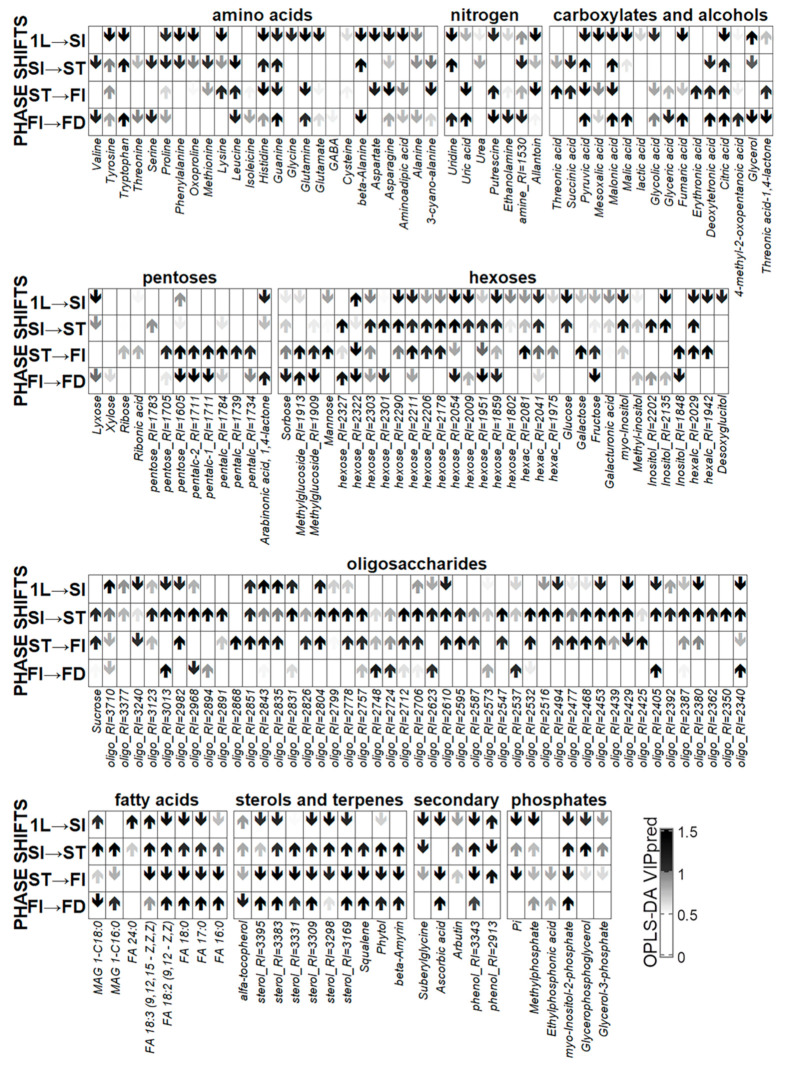
Metabolomic changes accompanying transfer between phases in +AM plants. Heatmap of OPLS-DA loadings (of predictive component), *p*. Up arrows refer to positive *p*, corresponding to level increased at the later phase.

Plants forming arbuscular mycorrhiza (+AM) reached the stooling initiation stage when non-inoculated plants (−AM) were still at the stage of two to three leaves (24 DAS). In order to determine how mycorrhization affects development from the 1LI to SI stages, the corresponding OPLS-DA model was designed. This included one orthogonal component, R^2^X = 0.60, R^2^Y = 0.96 (*p* = 0.02), Q^2^Y = 0.88, (*p* = 0.005); 31% were associated with the predictive component. As can be seen from Figure 9 and Figure S8, during the transition to stooling, a large number of metabolites, including fatty acids and sterols, as well as phosphates, decreased in +AM plants leaves. Similar processes occurred in −AM plants during this period (14–24 DAS), between the first and second/third leaf stages.

In order to compare the changes between the 1LI and SI stages in +AM and −AM plant metabolite profiles, a comparison of OPLS-DA models was fulfilled. It turned out that the proportions of variance associated with the predictive component did not differ in the case of +AM and −AM plants (about 30%). An SUS-plot graph (Figure S10A), wherein the metabolites are scattered in the loading space of the OPLS-DA models, showed that they have a lot in common and there was significant positive correlation between them (*ρ* = 0.50, *p* = 10^−16^). On the other hand, there were also differences that were mainly associated with metabolites, the accumulation of which was not noted during the transition to the stooling of AM plants (these metabolites are in the lower right sector of the graph). Among them were oligosaccharides and hexoses, as well as some carboxylates. An intensive increase in the content of the latter was observed in −AM plants during the transition to SI. Note that the accumulation of carboxylates was observed in +AM plants at the next ST stage, which coincides with SI in −AM plants in time.

#### 2.4.5. The Effect of Mycorrhization on the Leaf Metabolite Profile at the SI Stage

Changes in the metabolite profiles of leaves were also noted at the next stage of plant development. The OPLS-DA model, including one orthogonal component, showed that 28% of the variance was associated with the mycorrhization status (R^2^X = 0.57, R^2^Y = 0.99, *p* *=* 0.005; Q^2^Y = 0.91, *p* = 0.005). Factor loadings (Figure 7) showed that most of the metabolites were characterized by a decrease in the content level during mycorrhization. This dynamic was common to a number of sugars and their derivatives, both mono- and oligosaccharides. Perhaps it was due to the fact that the level of sugars in +AM plants increased sharply only at the next stage. Nonmycorrhized plants were characterized by a high content of a number of carboxylates, such as malate, citrate, fumarate, succinate, and malonate, among others. Additionally, in the leaves of plants of this variant, the content of some amino acids was higher, both proteinogenic and none. These were, among others, *β*-alanine, tryptophan, cyanoalanine, aspartate, asparagine, glycine, proline, and GABA. There was also an increase in the content of other nitrogen-containing compounds such as uridine, allantoin, urea, etc. The higher content of phosphorus-containing compounds—phosphate, ethylphosphonate, glycerophosphate—was a distinguishing feature of the +AM plants leaves. AM contributed to the accumulation of serine and methionine amino acids, as well as terpenes, phytol, and squalene.

Enrichment analysis revealed that mycorrhization was mainly associated with the repression of the Krebs cycle, the exchange of carboxylates and amino acids (Figure S8), and the decrease in the level of starch and hexose metabolism in the leaf. At the same time, an increase in the activity of sterol metabolism was observed.

To compare the effect of AM on plants in different phenophases, a SUS-plot was designed. The scattering of the predictive components of the OPLS-DA models in the loading space for the influence of AM at the 1LI and SI stages (Figure S10A) demonstrated that they differed significantly, but there was a significant correlation between them (*ρ* = 0.39, *p* = 10^−7^). The metabolites on which the AM influence differs were concentrated in the lower right sector of the graph. AM had a negative effect on the accumulation of these compounds. These were mainly hexoses and oligosaccharides.

#### 2.4.6. Metabolic Differences between the Stooling Initiation (SI) and Flowering Initiation (FI) Stages

##### The Changes in −AM Plants Metabolome

In non-mycorrhized (−AM) plants, two stages are identified during this period: from SI to SBI (29–45 DAS) and from SBI to FI (45–52 DAS). The designed OPLS-DA models included one orthogonal component for the stage from the SI to SBI: R^2^X = 0.65, R^2^Y = 0.98 (*p* = 0.01), Q^2^Y = 0.90, (*p* = 0.02); whereas for the stage of SBI and the FI, these indicators were equal to: R^2^X = 0.62, R^2^Y = 0.99 (*p* = 0.04), Q^2^Y = 0.98, (*p* = 0.04). For the first stage, 36% of the variance was associated with the predictive component. In the case of the second stage, the variance was 42%, which was more than for the previous stage, despite the fact that this stage was twice as short as the previous one. This may indicate a change in metabolism during the transition to flowering and is consistent with the above representation of the metabolomic profiles in a low-dimension space, wherein the FI observations were distant from the previous development stages (Figure 4 and Figure S6). Between 29 and 45 DAS in −AM plants, the content of fatty acids and sterols increased, the exchange of hexoses and oligosaccharides was activated, and the phosphate content increased, reaching values specific to +AM plants of the same age. At the same time, the content of many amino acids, nitrogen-containing compounds, and carboxylates decreased (Figure 8). As the enrichment analysis showed, at 29–45 DAS, changes affected a larger number of biochemical pathways compared to the previous stage (Figure S8).

Changes up to 45 DAS and further on to 52 DAS were quite different. This was evidenced by the graph of factor loadings of the last tested stages (Figure 8 and Figure S9). In contrast with the previous stage, the accumulation of pentoses occurred while the level of lipophilic compounds and phosphates decreased. There were numerous differences in the dynamics of the content of amino acids and carboxylates. At the same time, the accumulation of oligosaccharides and hexoses continued at the last stage, which was associated with a further increase in carbohydrate metabolism (Figure S8). The SUS plot graph clearly illustrates differences in the dynamics of metabolites between stages 29–45 DAS and 45–52 DAS (Figure S9). Between the values, a weak negative correlation was exhibited (*ρ* = −0.38, *p* = 10^−8^).

The differences in the changes at these stages became even more noticeable with enrichment analysis (Figure S8). As can be seen, in the period from SI to SBI, a number of pathways were repressed, including the metabolism of amino acids, TCA, and carboxylate exchange. On the other hand, the pathways of lipid synthesis (sterols and fatty acids), starch, and sugar metabolism were activated.

##### The Changes in +AM Plants Metabolome

For mycorrhized plants, two time periods from SI to ST stage (24–29 DAS) and from ST to FI (29–45 DAS) were distinguished. The OPLS-DA models generated included one orthogonal component. For the first segment, the parameters were: R^2^X = 0.49, R^2^Y = 0.95 (*p* = 0.005), Q^2^Y = 0.80, (*p* = 0.005); for the second segment, the parameters were: R^2^X = 0.44, R^2^Y = 0.99 (*p* = 0.01), Q^2^Y = 0.96, (*p* = 0.01). For the first period, 31% of the variance was associated with the predictive component, and for the second it was 33%. A similar depth of metabolomic alterations in periods that differ in duration indicated an inhomogeneity of the development and a strong shift in metabolic status during ST.

Significant changes from the stooling initiation to stooling stages were mainly characterized by a large-scale decrease in the content of many metabolites, primarily sugars, during the transition to SI (Figure 9 and Figure S8). This illustrated the metabolites scattering (Figure S9) in the loading space of the predictive components from the OPLS-DA models for 1L–SI and SI–ST. The opposite relationship was confirmed by a weak but reliable negative correlation (*ρ* = −0.17, *p* = 10^−10^). Thus, in mycorrhized plants, a decrease in the content of a large amount of sugars was first observed from 1L to SI (24 DAS) following a restoration of their concentration at ST (29 DAS). The advance of AM plant development compared to control −AM plants led to similar processes (although they were synchronous in both variants). However, these were observed in +AM plants at later stages of development.

At the next stage, from ST to FI, the content of hexoses and oligosaccharides continued to rise. During this period, there were also increases in the levels of pentoses. A similar process was observed during the transition to flowering of control −AM plants. During the transition to flowering of both +AM and control −AM plants, there was also a diminution in the level of sterols, possibly due to a decrease in their exchange level.

The similarity of changes in −AM and +AM plants occurring during the transition from SI to FI was visualized on the plot (Figure S11B). There was a strong reliable correlation between the loading values (*ρ* = 0.72, *p* = 10^−16^). Thus, the transition to flowering induces similar metabolic changes in the control (–AM) and during mycorrhization.

#### 2.4.7. The Effect of Mycorrhization on the Metabolite Profile of Leaves at Flowering Initiation (FI) Stage

Differences in the metabolite content at the FI stage were insignificant, a fact indicated by a smaller proportion of variance associated with the predictive component, namely 18%, despite the fact that when R^2^Y = 0.99, Q^2^Y was only 0.6 (for the predictive component, only ≈0.3). The significance of the obtained parameters was also low: *P*(Q^2^Y) = 0.16, *P*(R^2^Y) = 0.08.

Mycorrhization at this stage had a smaller effect than at previous ones. The effect of mycorrhization (Figure 7) included a decrease in the carboxylates level, which probably reflects the repression of TCA cycle and pyruvate metabolism. The level of sterols and fatty acids also decreased. In addition, the content of a number of hexoses and oligosaccharides was reduced during mycorrhization. Amino acids showed multidirectional trends. As in the previous stages, AM contributed to a greater accumulation of phosphate.

A comparison of AM influence at SI and FI stages (Figure S10B) showed that the loadings of the corresponding models, as in the previous case, differed, but the reliable correlation between them was weak (*ρ* = 0.27, *p* = 10^−4^). As in the previous case, the differences in the effect at SI and FI stages were mainly associated with the negative effect of AM on the level of a wide range of metabolites at the SI stage. 

#### 2.4.8. Metabolic Changes between Flowering Initiation (FI) and Flowering Development (FD) Stages

The revealed OPLS-DA model included one orthogonal component; the parameters were as follows: R^2^X = 0.55, R^2^Y = 0.94 (*p* = 0.04), Q^2^Y = 0.81, (*p* = 0.02); 19% of the variance was associated with the predictive component, which was lower compared to the previously analyzed transitional stages. As the loading diagram showed (Figure 9), the metabolic changes were virtually unrelated to the oligosaccharide content. This was quite unusual since their content changed quite rapidly in previous stages. Nitrogen exchange fluctuated noticeably during this period. The content of glutamine, uridine, guanine, and uric acid increased while serine, valine, leucine, putrescine, and ethanolamine decreased. It can also be noted that during the transition to fruiting, the content of carboxylates increased: malate, malonate, fumarate, and citrate. In addition, there was an increase in the content of sterols and fatty acids. In this context, there was a decrease in the level of monosaccharides, including fructose. Enrichment analysis (Figure S8) revealed that these changes were mainly related to the activation of the synthesis of lipophilic compounds (fatty acids and sterols), the activation of the TCA cycle and carboxylates in comparison with the repression of hexose and pentose exchange. An evaluation of the changes during the transition to flowering with the previous period revealed a significant alteration in the dynamics of metabolites, expressed in the inverse correlation of factor loadings corresponding to OPLS-DA models (Figure S9) (*ρ* = −0.45, *p* = 10^−10^).

#### 2.4.9. Comparison of the Effects of Mycorrhization and Plant Development on the Leaf Metabolome

In Figure S12, a SUS plot is shown, wherein metabolites are scattered in the space of loadings from two OPLS-DA models for comparison of −AM versus +AM plants at the SI stage (abscissa) and control −AM plants at the 2L and 3L stages (ordinate). As can be seen, the dependence between loadings was inverse (*ρ* = −0.44, *p* = 10^−16^), which indicates a lag in the metabolic development of +AM plants from −AM plants in the same phenological phase. This mainly concerned the delay in the accumulation of sugars described above. A comparison of the AM effect at the FI stage with previous metabolic changes also showed their negative dependence (*ρ* = −0.28, *p* = 10^−7^). This was consistent with the representation of metabolite profiles in a low-dimensional space, wherein the +AM profiles of plants shifted slightly towards the previous phase compared to the −AM plants profiles at the same development stage (Figure 4).

To sum up, we can conclude that AM not only accelerated development but also allow for the initiation of morphogenetic processes earlier, which is to say in metabolically more “young” plants.

### 2.5. Metabolite Networks

#### 2.5.1. The Dynamics of Metabolite Content during *M. lupulina* Development

**Figure 10:**
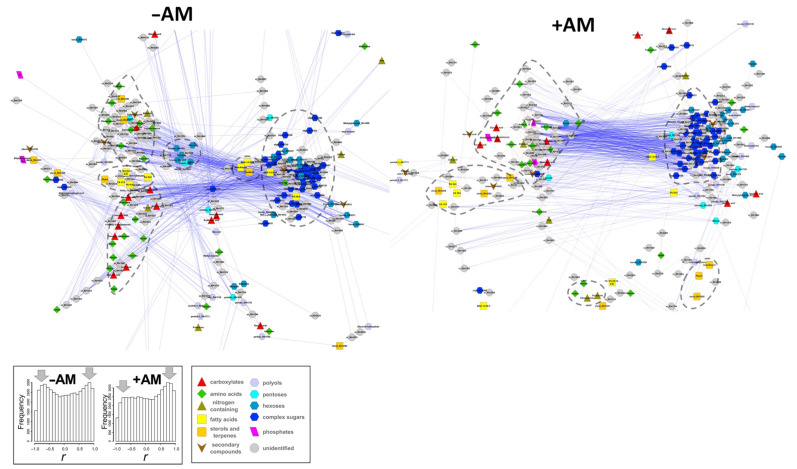
Comparative analysis of the correlations of metabolite levels. Mapping metabolites by correlations of their mean levels at different time points in the –AM and +AM plants. Nodes correspond to the metabolites, edges correspond to the significant *r* (*p* ≤ 0.01): red—positive, blue—negative. Positive correlations bring nodes together, negative ones push nodes apart as a physical force. Histograms illustrate differences in *r* frequencies under test (+AM) and control (−AM).

To identify the influence of AM on changes in intra-system connections during *M. lupulina* development, metabolites were mapped by correlations of average values of their concentrations. Figure 10 shows graphs where the nodes correspond to metabolites, and the edges correspond to a reliable correlation (*p* ≤ 0.01). A common feature for all graphs was the presence of a large dense group consisting mainly of carbohydrates, essentially complex sugars. The second cluster was connected to the first by multiple negative correlations. This cluster consisted of carboxylates, amino acids, and a small group of lipophilic compounds. Dense clusters were surrounded by a sparse network characterized by the presence of nitrogen and lipophilic compounds. It should be noted that in the case of –AM plants, amino acids and carboxylates formed distinctive groups. The behavior of monosaccharides also attracts attention. In the case of +AM plants, they were adjoined to the oligosaccharide cluster, but in the case of –AM, some of them formed small group clusters near the carboxylate-amino acid cluster. Analysis of the correlation values distribution for +AM and –AM plants (Figure 10) showed that they were far from the normal distribution. A special feature of the distributions was the high frequency of strong correlations. This presumably reflected the dynamics of metabolite pools, which were determined by rearrangements of metabolism with changes in development. Interestingly, the number of strong negative correlations decreased with AM. On the other hand, the disappearance of negative correlations may be the result of the fact that, for +AM, an increase in the pools of some compounds in this case did not require a decrease in the content of others. This could be a result of the fact that mycorrhization provides better growth conditions with a better supply of mineral nutrients to the plant and thus stimulates biosynthetic processes. In order to determine how the correlations in the content of metabolites differs due to mycorrhization, the correlation values were compared for all pairs of metabolites at +AM and control –AM. A positive correlation between them (*ρ* = 0.57, *p* = 10^−16^) was calculated. This suggests that the structure of correlations of the metabolites levels was quite similar in +AM and –AM plant development.

In addition, to determine how AM affects the connection of metabolites to each other during development, a graph was constructed wherein the metabolites were scattered in the space of the number of strong correlations (|r| ≥ 0.7) in –AM and +AM plants (Figure S13). In +AM groups, compounds containing phosphorus and the amino acid containing sulphur (methionine) became more associated with other metabolites, while the number of correlations between amines and carboxylates was greatly reduced. However, in common, the number of connections between +AM and –AM plants was similar, as evidenced by a positive correlation (*ρ* = 0.43, *p* = 10^−15^).

#### 2.5.2. Correlation of Metabolite Content

**Figure 11:**
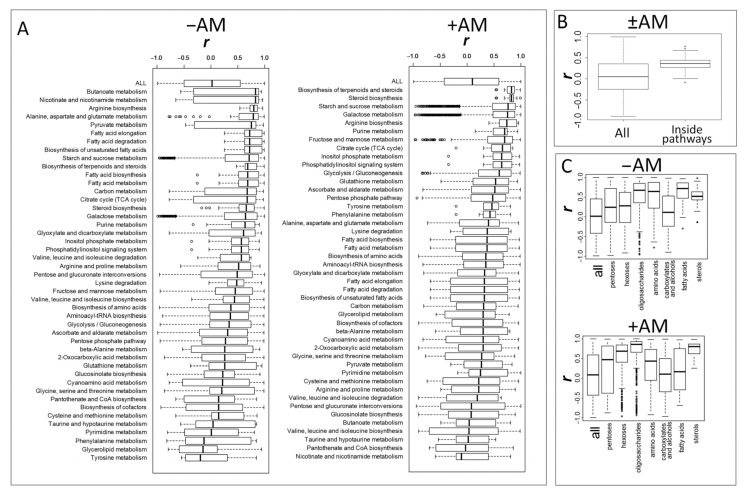
Relationship between biochemical pathway, chemical class, and metabolite level variations during *M. lupulina* plant development. Boxplots of Pearson’s correlations (“*r*”) between all pairs of metabolites (“All”) and for *r* counted for metabolites of the same KEGG pathway (**A**); for mean values of *r* inside each KEGG pathway (**B**); for *r* counted for metabolites of the same chemical class (**C**).

As mentioned earlier, compounds with a similar chemical nature and that are related metabolically are often located close to each other in the correlation network (Figure 10). This may indicate the influence of metabolic connections on the variability of the metabolome. To test this hypothesis, the average values of correlations within groups of metabolites gathered by a common biochemical pathway (KEGG) were considered (Figure 11A). It turned out that the metabolically related compounds were mainly associated with positive correlations. Different pathways showed various correlation interconnection of metabolites, depending on the status of mycorrhization. A strong positive relationship within the metabolic pathways was demonstrated by the boxplot, which shows the values of correlations of metabolites among themselves (the median was about 0 and the range of the values is from −1 to +1) and the average values of correlations for biochemical groups (Figure 11B,C). Note that they were strongly shifted towards positive values (Figure 11B). Therefore, metabolites within biochemical clusters are associated with positive correlations, even when their concentrations vary.

## 3. Discussion

### 3.1. Advantages of an Efficient Symbiotic Pair “M. lupulina + R. irregularis” Application

Most studies of the symbiotic relationships established between the host plant and the arbuscular mycorrhiza fungus are focused on the identification of mechanisms that increase the resistance of this above-organism system to the effects of unfavorable factors [39]. These include: a lack of water, an increase in temperature, an increase in the content of heavy metals, salinity [40], the intensity of the light mode [41], a lack of potassium [42] and nitrogen, and an excess [43] or lack of phosphorus [12,44,45]. For the most part, symbiosis is distinguished by a positive effect, but the intensity of the response varies over a wide range. This can be principally attributed to the genetic diversity of symbiotic pairs. The host plant’s responsiveness to the co-development with the mycobiont is quite important. Over comparative analysis, such a universal indicator as DAI (days after inoculation) is often used. For example, in the work [46], the interaction of *Funelliformis mosseae* and *Solanum lycopersicum* was evaluated on 7, 14, 21, and 28 DAI; and in the work [47], the contact of *Acaulospora tuberculata* and *Zea mays* was determined at 7, 14, 21, and 30 DAI. In these studies, the value of AM frequency (*F*) reached the highest values toward the end of the experiment (28–30 DAI). A study involving a responsive *Cucumis melo* plant with *Rhizophagus irregularis* revealed a significant *F* increase from 30 to 45 and 60 DAI [48], and in *Nicotianatabacum* with *Glomus etunicatum* from 20 to 40 DAI [49]. Thus, authors mainly considered relatively early stages of AM development and, due to the application of DAI, do not take into account the development stage of the host plant in the discussion of the results. However, even the above-listed plants differ in the time of start, and even in the existence, of such stages as leaf formation, stem formation (stooling), tillering, lateral branching, budding, and others.

Therefore, the study of the efficient AM formation mechanisms in this research was provided on leaves of the obligate mycotrophic *M. lupulina* MlS-1 line, responsive to inoculation with AM fungus *R. irregularis* under conditions of a low Pi level in the substrate. Furthermore, mycorrhization efficiency was simultaneously analyzed relative to the period after inoculation (DAI), and during *M. lupulina*’s development stages.

The analysis of the symbiotic development of *M. lupulina* plants showed an increase in the number and area of leaves, the plant height, the number of internodes, and fresh weight of aerial parts, which confirmed our earlier data [12,36,37,50]. This suggests that this line is highly responsive to different stages of the host plant’s development (Figure S2). It is noted that the stooling process (24–29 DAS) is accompanied by a temporal decrease in symbiotic efficiency of the number and area of leaves, as well as in the parameters of photosynthetic activity (Figure 3 and Figure S3). However, the next stage, SBI (38 DAS), can be considered as the “key” period for the formation of a highly efficient AM symbiosis between *M. lupulina* and *R. irregularis*. At the same time, the highest content of chlorophylls *a* and *b* in the leaves of mycorrhizal plants was detected during this period (Figure S4). A more detailed analysis of the AM effect on the intensity of the photosynthetic process of *M. lupulina* was carried out by examining photochemical indices. Thus, *Y(II)* (the amount of energy reaching the reaction centers of PSII) was higher (by 5.4%; *p* < 0.05) at 24 DAS and 38 DAS in *M. lupulina* plants with AM, which indicated a higher real photochemical activity of plant tissues with AM under conditions of a low Pi level. At later stages (45 DAS and 52 DAS), the effect was the opposite: *Y(II)* was lower (by 4–11%; *p* < 0.05) in mycorrhizal plants. The obtained data, in the mean, correspond to the previously detected increase in photosynthetic activity during the establishment of AM for various plant species, including various species of the Fabaceae family [51,52]. However, we can assume complicity of photosynthetic processes dynamics, which can be determined both by the own “needs” of the host plant and by the emerging structures of the AM fungus.

In summation, we suggest that the MlS-1 line of *M. lupulina* is responsive to the formation of AM under conditions of low phosphorus content in the soil. Over the course of establishing the symbiotic relationship, there are a number of changes in the host plant’s development and in biochemical processes. For example, the effect of mycorrhization on the growth and development of pea plants growing under the same conditions in a pair with the same symbiont differs significantly [34]. However, the mycorrhization efficiency in *M. lupulina* is not a constant parameter, and it changes during the development of the host plant, reaching a maximum at the SBI stage, 38 DAS.

### 3.2. The Effect of the AM Fungus R. irregularis on the Phosphorus Content in M. lupulina

The stimulating effect of AM on the plant is associated primarily with the benefits of mineral nutrition intensification. In the present study, mycorrhization by the AM fungus *R. irregularis* led to a significant increase in the content of Pi (free phosphate) and glycerophosphoglycerol in the *M. lupulina* leaves (Figure 1). An increase in the Pi content in leaves under the influence of AM has been described in various plant species [33,52,53,54]. However, this phosphorus-caused “growth effect” of AM is not always detected. For example, some studies have demonstrated that the differences in growth intensity between inoculated and control (without a symbiont) plants may be insignificant [33]. Perhaps the lack of efficiency depended on the concentration of minerals in the soil, which were not enough for the development of AM. On the contrary, there were no differences in the mass of shoots at high Pi [26]. In the case of *Plantago major*, mycorrhization had practically no effect on the growth of the biomass of the aboveground parts, but there was an increase in the Pi content in the leaves at late development stages [52].

### 3.3. M. lupulina Leaves Metabolite Profiling 

#### 3.3.1. General Metabolic Patterns in *M. lupulina* Leaves Development

Metabolomic analysis revealed the distinctiveness of plants at different growth phases. The main feature of development at the metabolomic level is strong nonlinearity (Figure 4 and Figure S6). The absence of monotonicity and irregularity of biochemical and physiological changes during growth of biological systems are common [34,35,55]. An important feature of these processes is the different proximity of phases that mirror the scale of metabolic shifts. It can be noted that the most extensive metabolic rearrangements accompany the transition to stooling (Figure 4 and Figure S6).

Metabolomic alterations are accompanied by drastic physiological changes owing to mycorrhization. The transition to stooling is marked by an explosive increase in AM efficiency (Figure S2). It is accompanied by a dramatic growth in *NPQ* and a decrease in *Y(II)* (Figure 3). There was also a rapid increase in the ratio of the carotenoid sum to the sum of chlorophylls (Figure S5). Then, *NPQ* decreased and *Y(II)* increased in *M. lupulina* AM plants in the SBI stage vs. the noninoculated control (Figure 3). These data are consistent with results in *Salvia fruticosa* with *R. irregularis* [56]. Thus, it was during the stooling process that the beginning of global metabolic rearrangements occurred in AM plants, including the photosynthetic activity, biochemical status, and interaction with the fungus.

The second period of large metabolic perturbations is a flowering initiation (Figure 4 and Figure S6). Both +AM and –AM plants at the flowering stage were characterized by a low ratio of carotenoids to chlorophylls (Figure S5) relative to the previous stages, and an increase in the photosystem II (PS II) efficiency level. Moreover, the late development stages of –AM plants were characterized by an increase in the *NPQ* level more intense than in +AM plants. Similarly, in *Salvia fruticosa*, *Y(II)* was higher, and *NPQ* was lower in plants inoculated with *R. irregularis* at 133 DAS vs. –AM plants [56].

#### 3.3.2. The Effect of the AM Fungus *R. irregularis* on the *M. lupulina* Leaves Metabolite Profile

The development of close symbiotic interaction between the participants of AM symbiosis involves an intensive exchange of metabolites, primarily carbohydrates, nitrogen- and phosphorus-containing compounds, etc. In this regard, a system analysis of changes in the leaf metabolite profiles during the formation of AM-symbiosis is of particular interest.

The development of modern methods of metabolite detection, including gas chromatography coupled with mass spectrometry, permits researchers to perform metabolic profiling in the comparative analysis of leaves in a number of model species: *Anadenanthera colubrina, Artemisia annua, Castanospermum australe, Catharanthus roseus, Citrus tangerina, Cynara cardunculus, Fortunella margarita, Hordeum vulgare, Hypericum perforatum, Libidibia ferrea, Lotus japonicus, M. truncatula, Mentha viridis, Origanum vulgare, Plantago lanceolata, P. major, Plectranthus amboinicus, Rosmarinus officinalis, Stevia rebaudiana, Ocimum basilicum, Vitis vinifera* (Rev. [27]), and *Glycine max* [57]. The accumulated data indicate a significant AM influence on both the primary and secondary metabolism of plants. However, it is interesting to note that the biochemical changes are extremely species-specific, even when closely related plant species are compared [31,32,33,58,59]. Thus, the question arises of the validity of applying the results of plant metabolic spectra analysis over different symbiotic pairs [27,33].

Previously, a comparative leaf metabolic analysis of *Lotus japonicus* cv. *Gifu* plants inoculated with the BEG 12 *Glomus mosseae* strain (according to the new classification: *Funneliformis mosseae*; [60]) at late development stages of the host-plant DAI against a –AM variant was performed at 70, 77, and 84 DAS [61]. PLS-DA revealed some similarities between the control and the variant with the mycosymbiont. The analysis of factor loadings separating mycorrhizal and non-mycorrhizal plants still allowed us to determine the main set of metabolites specific for each of the variants. In general, the results showed a negative systemic effect of AM colonization on the content of organic acids that play a role in the main catabolism pathways and in amino acid metabolism [61]. However, the importance of metabolic profiling is supported by the data of a recent study conducted on the leaves of a plant that is unresponsive to AM, *Pisum sativum*. A significant dependence of the metabolic profile on the rate of mycorrhization of peas was revealed [34]. It was shown that the development of AM with *P. sativum* significantly prolonged the active phase of the host plant’s metabolism in the absence of a growth response. At the metabolomic level, this could be recognized as a delay in development and the delayed aging of mycorrhizal plants.

The effect of AM on the metabolome increased during the beginning of stooling, when the AM efficiency started to increase (Figure S2), and the development of symbiosis interfered with photosynthetic activity. The PSII efficiency values and the chlorophyll content in +AM plants became higher and *NPQ* lower than in –AM plants (Figure 3 and Figure S4). Then, during the transition to flowering, the metabolomic differences of +AM and –AM plants were reduced. The AM efficiency during this period also decreased.

A very particular phenomenon was discovered during this investigation. Mycorrhized plants moved to the next phenological stage earlier than control (–AM); however, its metabolic status did not yet pass through all the biochemical changes that took place in the corresponding stages of plants without AM. Some similarity can also be observed with respect to the dynamics of physiological parameters, in particular, the dynamics of growth (Figure 2), changes in the *Y(II)* and *NPQ* indicators (Figure 3). For example, at 38 DAS, the *NPQ* level was decreased in both +AM and –AM plants, followed by a sharp increase to 45 DAS. However, in the case of the +AM variant, this period lasted from SBI to FI, and in –AM plants, it continued from ST to SBI. A *Y (II)* level decrease was noted in both studied variants at 29 DAS (Figure 3), followed by an increase at 45 DAS. However, the +AM SI stage at 24 DAS maintained a *Y(II)* value that was still high, whereas the –AM SI stage corresponded to 29 DAS when the value of *Y(II)* had already decreased. The reverse was the case when the +AM ST stage stood at 29 DAS: a low *Y(II)* level, whereas the –AM ST stage corresponded to 38 DAS when the value of this parameter was high again. Thus, the values of a number of analyzed physiological and metabolic parameters in +AM plants were similar to those in –AM plants, but at the previous phenological phase.

Consequently, the metabolic profiling of another legume, *M. lupulina*, characterized by a high efficiency of interaction with the AM fungus, is of particular interest. The obtained data indicate that the *M. lupulina* leaf metabolite spectrum consists of more than 300 compounds (Table S1). They represent a large variety of carbohydrates and their derivatives, typical for plant leaves [34,62]. The subsequent development of the host plant and the symbiotic pair is clearly characterized by significant changes in the metabolic profiles and/or the metabolite content. An initial comparison of the data set using the principal component method demonstrated that the profiles are grouped according to the development stage (Figure S6). The obtained data proved that development at the metabolic level is a complex nonlinear and nonuniform process. However, it remains unclear which of the factors or their combination determines these changes.

### 3.4. M. lupulina Mycorrhization, Phenophase and Age: Factors Interference 

The assessment of the *R. irregularis* influence on the development of *M. lupulina* was considered as a sequential change of developmental stages. The processes occurring in the host plant were supposed to be a result of the sequential activation of its own developmental program, and as a result of its modification due to interaction with the AM fungus. 

This study shows that plants without AM significantly lagged behind mycorrhizal plants in development (Figure 2). Therefore, to correctly determine the “time points” for comparison of +AM and –AM plants, it is necessary to determine what better characterizes the plant’s biochemical status: calendar age (DAS/DAI) or phenophase. The obtained data indicate that the metabolome is associated more with the stage of development than the age of the plant (Figure 6). However, it should be noted that most of the identified processes occur synchronously during the formation of AM and in its absence. Perhaps they serve to mediate the possibility of the transition of mycorrhizal *M. lupulina* plants to the next phenophase at a relatively “younger metabolic age” (Figure 6). The following are some examples.

The evaluation of changes in the metabolic profiles of *M. lupulina* under the influence of the AM fungus *R. irregularis* revealed a number of features already at the 1L stage. In +AM plant leaves, a decrease in the content of compounds metabolically associated with the TCA cycle was observed (Figure 7). Previously, a similar process was shown for the roots of mycorrhizal *M. truncatula* [26]. This reaction was typical for the *M. truncatula* leaf metabolome [33] and many other dicotyledonous plants [33,34,52,61]. The revealed effect of mycorrhiza was probably due to the negative effect of symbiosis on central catabolism as a result of increased carbon transport from the host plant to the AM fungus. In addition, carbon redistribution between catabolism and protein synthesis may occur as a result of changes in the nitrogen–phosphorus balance [33,61]. It is interesting that in previous studies on other objects, this effect was demonstrated at late stages, but in our investigation on *M. lupulina*, it clearly appeared already at the 1L stage. This pattern can be explained by a high level of responsiveness to mycorrhization in the *M. lupulina* line expressed in a more intense effect of AM on the formation of the aerial organs biomass, an effect that was not observed in previous studies [33]. Note that the content of some amino acids in the *M. lupulina* leaves increased during mycorrhization (Figure 7). Previously, a similar phenomenon was detected in mycorrhizal plants *Plantago lanceolata, P. major, Veronica chamaedrys, M. truncatula*, and *Poa annua* [33]. In *M. truncatula*, the activation of plastid metabolism was shown in response to mycorrhization. It was expressed in an increase in the level of a number of amino acids, as well as some fatty acids [26]. The last study was the most detailed in terms of the duration of the experiment: analyses were performed at seven time points weekly from 14 to 56 DAI [26]. A significant increase in the frequency of AM (*F*) was shown from 27% to 88% during 21–35 DAI. However, changes in the number of arbuscules and vesicles in the roots, as well as changes in AM efficiency, were not analyzed during this long-term experiment. However, for the first time, AM-specific fatty acids, such as palmitvaccenic and vaccenic acids, were considered as markers of colonization by the AM fungus *Rhizophagus sp.* [26].

Further analysis was to determine changes at the SI stage and during the transfer of the phenophase from 3LI to SI. A negative relationship between the action of mycorrhiza and developmental changes in the metabolome was revealed (Figure S12). At the SI stage, when this effect was most pronounced, this dependence was primarily associated with an earlier transition of +AM plants to SI, even before the accumulation of sugars began. This led to a lower level of sugars in the leaves of mycorrhizal plants compared to plants that do not form AM at the SI stage. The question arises: what mechanisms are behind the acceleration of +AM plant development? What is the role of the large-scale accumulation of sugars observed in plants during the transition to stooling? What is the relationship of this process with mycorrhization if it is the case that +AM plants pass to stooling without it and the accumulation of sugars is observed already in the process of stooling?

It can be assumed that the effect of mycorrhization was due to differences in metabolic networks that were in demand at different stages of host-plant development (for example, in the SI and FI stages). However, along with this, it is necessary to take into account the constant change in the mutualistic interaction, since both the costs of the plant (photoassimilates) and the benefits (Pi) change during the AM interaction [52]. A similar pattern was observed in analysis of the AM effect on the *P. sativum* [34] and *Senecio jacobaea* [28] leaf metabolome. For the *M. truncatula* roots, the dominant role of host plant growth in the determination of the metabolome in relation to the regulatory value of AM symbiosis was established [26]. This once again confirms the priority of the host plant’s ontogenetic program, including at the metabolomic level.

At the stooling stage (ST), the vast majority of metabolites were characterized by a lower level of content in the +AM variant compared to non-mycorrhizal plants (Figure 7). Similarly, it was noted that the number of metabolites whose level decreased in the leaves of mycorrhizal *P. major* was several times higher than the number of metabolites whose content increased. Perhaps this is due to an increase in the outflow of photoassimilates to the growing AM fungus [52]. The intensity of this outflow can reach 20% of the host plant photoassimilates [63]. This may explain the revealed decrease in the level of oligosaccharides and hexoses at this stage in +AM plants. Interestingly, the increase in the sugar accumulation was observed in their development later and coincides in time with that in plants without AM.

The transition to flowering and fruiting is the final stage not only of the development of the host plant but also of the entire plant-microbial system as a whole. As expected, during this period, there was a weaker effect of AM fungus on both the metabolic spectrum and the dynamics of the content of individual compounds (Figure S6B).

### 3.5. The Relationship of the Topology of Metabolic Pathways with the Correlation of the Metabolite Content

An important aspect of omics research is the analysis of the relationships between the components of biological systems. The correlation can be used to assess how closely metabolites are related to each other [64]. This is possible because they behave in a consistent manner and changes caused by external or internal factors lead to a modification of the state of the entire system, i.e., to a change in the connections between the metabolites, which will be reflected in their correlation patterns [65,66,67]. It has been shown that it relates specifically to organs and tissues [68,69]; to the genotype, including point mutations [68]; and to environmental conditions [70,71]. 

An analysis of biochemical networks indicates that such a network is scale-free and was proposed to characterize the Internet and social networks. In scale-free networks, the degrees of nodes (the number of connections) are distributed according to a power law. Such networks are characterized by heterogeneity and the presence of a small number of nodes with a high number of connections. Such nodes are called hubs. It is believed that hubs can play an important biological role [72,73]. Interestingly, in contrast to non-biological networks, where the number of node connections is fixed and with an increase in the number of them, the network diameter increases in metabolic networks and the diameter of the networks may not differ in more complex and simple organisms owing to the fact that the number of reactions in which the substrate participates may increase with the complexity of the organism [72]. 

Many different approaches exist for evaluating the metabolite relationships [67,74,75]. The correlation coefficients are basic ones. To analyze the effect of mycorrhization on the structure of the relationships of dynamic changes in the levels of metabolites during *M. lupulina* development, they were mapped by reliable (*p* < 0.01) correlations of their average content (Figure 10). The data of the resulting network corresponded to a scale-free organization with its inherent heterogeneity. The diameter of the analyzed networks, both with and without mycorrhization, was 14. In the networks we built, the average path lengths (4.49 for –AM and 4.72 for +AM) as well as other global characteristics of the networks did not differ significantly. In our case, during mycorrhization, the number of strong negative correlations decreased and positive ones increased. A decrease in the number of strong negative correlations may be the result of the fact that at +AM, an increase in the pools of some compounds did not require a decrease in the content of others. It resulted in positive correlation prevalence. The reason for this may be more favorable growth conditions provided by mycorrhization.

The prevalence of positive correlations was also observed in other biological objects, such as the roots and leaves of Arabidopsis [68], human tumor cell cultures [71], and microalgae cultures [55,76]. The reason for the high positive correlation may be proximity to equilibrium and a high level of metabolic flow. Such a quasi-stationary state leads to indirect system-wide correlations between remote metabolites [65,77]. Correlation is a property of a system, and not of a single reaction, enzyme, or metabolite since they are the result of a combination of all reactions and regulatory processes [65,66,67]. Another feature of the distributions in our networks is the high frequency of strong correlations. Usually, most of the correlations in biological systems have moderate values [65]. Perhaps the reason for the rising of correlations is the strong influence of the developmental program on the dynamics of metabolic profiles.

At the next step, we compared the correlation values for all pairs of metabolites, with +AM and –AM variants. The positive correlation (*ρ* = 0.57, *p* = 10^−16^) indicated that the structure of the metabolite level correlations was quite similar in the development of both +AM and –AM plants. The conservativeness of correlations under changing conditions has been encountered before. Thus, when the *E. coli* cultivation conditions changed, many correlations were conservative, for example, those associated with the TCA cycle [68]. As can be seen from Figure 10, in the case of *M. lupulina*, clusters associated primarily with complex sugars and amino acids were preserved. The formation of clusters and regions according to the chemical nature and pathways of biosynthesis was also observed in the other model system, “*Pisum sativum* + *R. irregularis*” [34]. The study of the roots and aerial parts of Arabidopsis and potatoes showed that there was also an amino acid cluster [69]. Fatty acids and terpene derivatives also form small groups, some of which are located on the periphery of the network. The largest cluster was formed by sugars. There was also an area that unites carboxylates, among which there were many intermediates associated with TCA cycle, and some amino acids, which may be the result of their metabolic connection.

The following question then arises: does a relationship between the structure of a biochemical network and variation in metabolite levels exist? To answer that, distributions of correlations within sets of metabolites for certain biochemical pathways (extracted from KEGG) were analysed (Figure 11A,B). In addition, correlations in metabolite sets, combined by the molecular similarity, were also calculated (Figure 11C).

In our study for *M. lupulina*, it was revealed that the values of correlations within biochemical groups are significantly shifted in a positive direction. The reason for the increased correlations within the biochemical pathways may be the restrictions imposed by the sequences of biochemical reactions on the possibility of independent changes in the levels of metabolites.

Different groups of metabolites diverged significantly in the variation of correlations. At the same time, the status of mycorrhization significantly affected the distribution of correlations within groups. A high level of correlation connectivity was demonstrated by a number of highly specialized pathways, including the exchange of fatty acids, lipids, and sugar metabolism. Note that during mycorrhization, the binding of fatty acids was much lower. Similar dynamics were found for amino acids. For some groups, the distribution of correlations was characterized by a distribution that did not differ from the general range. Such segments of the metabolic network and chemical classes can be considered as “intersections” of several metabolic pathways with stronger internal regulatory connections—for example, carboxylates, which are intermediates of many processes.

### 3.6. M. lupulina Mycorrhization, Phenophase and Growth Duration: Distances Covered

**Figure 12:**
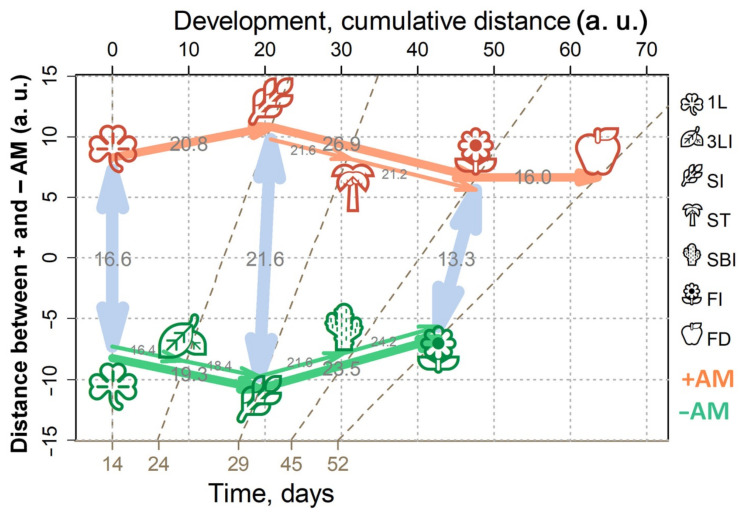
The scales of metabolic changes during development with or without mycorrhization. Gray numbers—distances (a.u.) in the Euclidean space of normalized metabolite levels.

In conclusion, we will focus on such an indicator as the duration and distance of transitions between different phenophases. Earlier, we assumed that the AM-symbiosis formation program interferes with the developmental program (the host-plant development program), which leads to the fact that the effect of mycorrhization at different phenological phases differs both qualitatively and quantitatively. As it turned out, the differences between –AM and +AM plants first increased and then decreased up to the flowering stage (Figure 4 and Figure 12 and Figure S6B). Perhaps the decrease in the action of AM during the transition to flowering is explained by the rigid determination of this stage since it is the most important for the realization of reproductive function. On the other hand, according to the literature data for some plant species, the effect of AM was more pronounced precisely at the late stages [26,52], which indicates the possibility of implementing various scenarios of interaction between the fungus and the host plant depending on the genotype.

To assess the quantitative changes in the metabolite profiles that occur during mycorrhization and development, visualization of the “distances traveled” in the space of the metabolite content levels was carried out (Figure 12). The purpose of this approach was to demonstrate the correlation of quantitative changes, and, consequently, the “work” carried out by the plant in the ontogenesis process and during the development of AM symbiosis. Figure 12 shows the cumulative distance between the three general stages of development: the first leaf (1L), the stooling initiation (SI, beginning of stem development), and the flowering initiation (FI) along the abscissa axis (Figure 12). In Figure 12, all stages are connected by heavy lines. The segments within these distances are represented not by absolute length but by proportional fragments of the section within which they are located (thin lines). Based on these results, several important hypotheses can be formulated. Firstly, the distances that +AM and –AM plants “passed” were very close (on similar segments in the phenophase). However, mycorrhizal plants passed a slightly longer distance. This was due to the greater nonlinearity of their development, which is perfectly illustrated by the representation of profiles in a low-dimension space (Figure 4 and Figure S6). Consequently, mycorrhization caused a complication of the development of the host plant. Secondly, the distances between time points grew until FI. This was especially clearly observed in the variant of control plants. Perhaps this was connected with more significant rearrangements in the *M. lupulina* metabolic profile. Thirdly, there was a high nonlinearity of the processes (Figure 4 and Figure S6), expressed in the non-additivity of the distances covered. So, for example, within the transition of –AM plants from SI to FI with a length of 23.5 a.u., two stages of 21.6 and 24.2 a.u. were nested. Finally, the distances in the initial space between +AM and control –AM plants, although lower than between the stages, were insignificant. At the same time, as we have already observed, the strength of changes between different stages and the strength of the influence of mycorrhization were unequal in different periods of plant growth (see also Figure 4 and Figure 7 and Figure S6).

The analysis of the configuration of the influence of mycorrhization and phenophase using PCA and MDS (especially in Euclidean space) suggests that the revealed differences may be due to a more narrowly directed effect of mycorrhization compared to changes occurring as a result of a larger-scale influence of ontogenetic programs (change of phenophases).

## 4. Materials and Methods

### 4.1. Biomaterials: Model Plant and Test of Arbuscular Mycorrhizal Fungus

The *Medicago lupulina* model plant was used to study AM-mediated metabolic alterations in leaves. The selected MlS-1 line from cultivar-population VIK32 was used in this study. Plants of this line showed the signs of dwarfism in the absence of inoculation with AM fungus at a low level of available phosphorus in the soil (Pi) [12,36,37,50]. 

The *Rhizophagus irregularis* strain RCAM00320 was obtained from the collection of Laboratory of Ecology of Symbiotic and Associative Rhizobacteria at the All-Russia Research Institute for Agricultural Microbiology (ARRIAM). The strain, previously known as the *Glomus intraradices* Shenck&Smith strain CIAM8) used for inoculation, was previously characterized as a fungus forming highly effective AM symbioses with a majority of agricultural crops [12,36,37,78,79,80,81,82]. Accurate identification of the strain was provided [83]. Since AM fungi are obligate symbionts, the strain was grown with Swedish ivy (*Plectranthus australis* R. Br. = *P. verticillatus* (L.f.) Druce, *P. nummularius* Briq.) under standard conditions in our Laboratory (fungal inoculum preparation is described in Figure S14).

### 4.2. Experimental Design and Collection of Plant Material

*M. lupulina* seeds were surface sterilized as follows: scarification for 5 min in concentrated H_2_SO_4_ and then multifold rinsed with sterile water for 120 min. Sterilized *M. lupulina* seeds were put on sterile paper filter in Petri dishes for 1 day at +4 °C for stratification. Then, *M. lupulina* seeds were germinated for 2 days at +27 °C in the darkness. Seedlings of equal size were planted with the growth substrate described in ‘plant growth conditions’. Half of the plants were inoculated with fungal inoculum before planting (+AM); the other half was not treated by fungal inoculum as control (–AM). Twelve plants per treatment were taken for each biochemical and microscopic analyses at random at each stage of plant development. The plants were removed from the soil and their root systems were thoroughly washed. The plant height; fresh weight of plant roots and aerial parts; and the number of leaves, internodes, and peduncles with buds, flowers, and fruits were determined. After measuring the growth parameters, the youngest fully developed leaf from each analyzed plant was cut off. Leaves from 8 plants were allocated to a single biological replicate, weighed and frozen in liquid nitrogen in a 2 mL Eppendorf Safe-Lock tube, and then stored at −80 °C. At least three biological replicates for each time point were collected for biochemical analysis. For arbuscular mycorrhiza analysis, fragments of roots were collected individually from each plant and were dried at room temperature.

### 4.3. Plant Growth Conditions

Four seedlings were planted in one pot filled with 210 g of a soil:sand (2:1) mixture (agrochemical soil characteristics are presented in Table S3; P content available to plants was calculated using the Kirsanov reaction and was very low according to [84], since one was <25 mg/kg). The substrate for growing was autoclaved at 134 °C, 2 atm. for 1 h with repeated autoclaving after 2 days. Such a procedure prevents mycorrhization. The microvegetative method provided optimal conditions for AM development and allowed to avoid spontaneous infection by rhizobia and other symbiotic microorganisms. The first datapoint was collected at the 14th day after sowing and inoculation. Further testing was carried out at the key stages of plant development. Biochemical and microscopic analyses of plants were performed on the 14th, 21st, 24th, 29th, 38th, 45th, and 52nd day after sowing (DAS) and inoculation (DAI = DAS in our research) (Table 1).

**Table 1:** The plant development stages in *Medicago lupulina* under the condition of a low phosphorus level in substrate and with/without AM inoculation.

Analysis No	Day after Sowing (DAS)	Host Plant Development Stage	
		−AM	+AM
1	14	1st leaf development (1L)	1st leaf development (1L)
2	21	2nd leaf initiation (2LI)	2nd leaf development, 3rd leaf initiation (3LI)
3	24	2nd leaf development, 3rd leaf initiation (3LI)	stooling initiation, 3rd leaf (SI)
4	29	stooling initiation, 3rd leaf (SI)	4th leaf, stooling (ST, stem formation)
5	38	4th leaf, stooling (ST)	5th leaf development, shoot branching initiation (SBI)
6	45	5th leaf development, shoot branching initiation (SBI)	6–7th leaf development, shoot branching, flowering initiation (FI)
7	52	6–7th leaf development, shoot branching, flowering initiation (FI)	8–9th leaf, green pod initiation, flowering development (FD)

Note: “−AM” is the variant without AM fungus inoculation, “+AM” is variant with inoculation with *R. irregularis* AM fungus.

### 4.4. Evaluation of Mycorrhization Parameters

The method of maceration and staining of root samples, designed by J.M. Phillips and D.S. Heyman for the estimation of AM infection in the roots of leguminous plants [85], was used for the microscopic analysis of AM development, including staining with trypan blue in solution containing 10% lactic acid, glycerine, distilled water, and “Trypan blue” dye in a ratio of 62 mL: 62 mL: 875 mL: 0.3 g, respectively [36]. For microscopic analysis of AM structures in crushed preparation: dried, macerated, and cut (10 mm long) *M. lupulina* roots for analysis by light microscopy (Mikmed-2 var.2 microscope, LOMO, Russia; characteristics: eyepiece 10, lens 10, front lens in the flange of the binocular adjustment 1.0). The estimated length of the microscope field of view was 1.69 mm. Subsequently, mycorrhization indices were calculated [86]: *F* (the frequency of mycorrhizal infection in the roots), *M* and *m* (the intensity of AM in the roots and mycorrhized parts of roots, respectively), *A* and *a* (the abundance of arbuscules in the roots and mycorrhized parts of roots, respectively), and *B* and *b* (the abundance of arbuscules in the roots and mycorrhized parts of roots, respectively). The microscopic analysis of AM development was provided by computer program for calculating the mycorrhization indices of plant roots developed by A.P. Yurkov et al. [87]. The minimum biological replication in each of studied variants was eight.

### 4.5. Evaluation of AM Symbiotic Efficiency

The efficiency (mycorrhizal growth response) was calculated for parameters of productivity of the plants using Odum’s formula:

E = ([+AM] − [−AM]) × 100%/[−AM](1)

where E is the AM symbiotic efficiency, [+AM] is the value of productivity parameters in mycorrhized plant (for example, fresh weight), and [–AM] is the value of productivity parameters in plants without AM.

### 4.6. Evaluation of Leaf Photochemical Activity and Pigment Content

The central leaf blade in the youngest completely formed leaf was measured by the fluorescence method to evaluate the leaf photochemical activity. Plants (three biological repeats for each variant) in pots were placed in a light-tight chamber and pre-adapted to darkness for 15 min before the measurements. The kinetics of the chlorophyll a fluorescence induction was acquired at room temperature by pulse amplitude modulation (PAM) fluorometric analysis using a portable chlorophyll fluorometer PAM-2500 (Heinz Walz GmbH, Effeltrich, Germany). To secure the leaflets, a 2030-B clamp equipped with a quantum and temperature sensor was used. The chlorophylls in PS II were excited by the light-emitting diode characterized by the wavelength of 750 nm. Fluorescence detection was performed using a PIN photodiode protected by a "long-pass" filter characterized by the wavelength of 715 nm and 50% transmission. Using PAMWin-3 Software and Instruction manual for PAM-2500 [88], the following fluorescent parameters were obtained: *Fv*/*Fm*, the maximum PSII photochemical efficiency in the darkness-adapted state [89]; *Y(II)*, the effective quantum yield of photochemical energy conversion in PSII [90]; *ETRmax*, the maximum electron transport rate at light saturation; *I_K_*, minimum saturating irradiance; *qP*, the coefficient of photochemical quenching of chlorophyll fluorescence; and *qN*, the coefficient of non-photochemical quenching of chlorophyll fluorescence [91]. The leaflet area was calculated using Fovea Pro v. 4.0 for Adobe Photoshop [92].

Leaf pigment analysis was conducted with 3 biological repetitions. The leaf samples (0.01 g) were ground three times for 2 min in 2 mL microtubes with 3 metal balls 3 mm in diameter in liquid nitrogen by using a Tissue Lyser LT (Qiagen, Hilden, Germany) bead mill at a 50 hits s−1 frequency. The pigments were quantitatively extracted with methanol as described by Smolikova et al. [93]. The absorption spectra of the extracts were measured at 470.0, 652.4, and 665.2 nm in quartz cuvettes with a 1 cm light path (Reachim, St. Petersburg, Russia) by using a UV/Vis spectrophotometer Spekol 1300 (Analytik Jena AG, Jena, Germany). The chlorophyll and carotenoid contents were calculated as recommended by Lichtenthaler [94] and Lichtenthaler and Buschmann [95] and normalized to fresh weights.

### 4.7. GC-MS Analysis 

Leaves were sampled at 14, 24, 29, 45, and 52 DAS. Samples were processed as previously described [82]: 100 mg of samples were immediately frozen with liquid nitrogen. Then samples were grounded with bead mill (MM 400, Retsch, Haan, Germany) and subjected to a single-stage extraction with a 2 mL methanol:chloroform:water mixture (5:2:1) with continuous shaking 900 rpm at 4 °C in a thermoshaker (BioSan TS-100C, Riga, Latvia). Tissue debris was removed by centrifugation at 12,000× *g* for 10 min at 4 °C. The supernatant was collected and evaporated in a vacuum concentrator (CentriVap, Labconco, Kansas City, MO, USA). For GC-MS analysis, dried material was dissolved in pyridine with the tricosane (normal hydrocarbon) as internal standard. The samples were then supplied with the silylating agent BSTFA:TMCS 99:1 (Sigma-Aldrich, Saint Louis, MO, USA) and derivatizated at 90 °C for 20 min.

GC-MS analysis was performed with an Agilent 5860 chromatograph equipped with a DB-5 MS capillary column and coupled with an Agilent 5975 quadropole mass selective detector under control of Agilent ChemStation software (Agilent Technologies, Santa Clara, CA, USA). The helium flow rate was 1 mL/min. The inlet temperature was 250 °C at splitless mode. The column temperature started from 70 °C and increased up to 320 °C by 5 °C per min. Electron impact ionization was performed at 70 V and an ion source temperature of 230 °C.

The analysis of the GC-MS data was processed using the PARADISe software (Department of Food Science Faculty of Science, University of Copenhagen, Denmark; [96]) coupled with NIST MS Search (National Institute of Standards and Technology (NIST), USA). Moreover, for deconvolution and metabolite identification, the AMDIS (Automated Mass Spectral Deconvolution and Identification System, NIST, Gaithersburg, Maryland, USA) was used. Compounds were identified by obtained mass-spectra and Kovats retention indices using libraries: NIST2010, Golm Metabolome Database (GMD; [97]), and an in-house library. The peak was referred to the identified metabolite when the match factor was >800. Additionally, the Kovats retention indices were used for identification. When one compound was represented as several peaks (isomers or several TMS forms), its areas were summed.

### 4.8. Statistical Analysis

All data on plant growth, mycorrhiza development, pigment accumulation, and chlorophyll fluorescence were processed with one-way ANOVA. The SPSS 12.0 package (SPSS Inc Chicago, IL, USA) was used for ANOVA. The Tukey’s HSD test as a post hoc test was used to compare differences in mycorrhization parameters at different stages of plant development. All data were expressed as the mean ± standard error. The differences were considered as significant at the confidence level of *p* ≤ 0.05.

Statistical analyses of the metabolomic data were processed in the R language environment 3.6.3 [98]. For quantitative interpretation, the peak area was normalized against the area of the internal standard (tricosane). Data were normalized against the sample median. Outlying values were excluded on the basis of Dixon’s test in outliers package [99]. The data were log_2_-transformed and standardized. If a compound was not detected in a sample but was present in the other replicates, it was considered as a technical error and imputed by KNN (k-nearest neighbors) with the impute R package [100].

A heatmap was made with ComplexHeatmap [101]. Principal component analysis (PCA) was performed with *pcaMethods* [102]. The random forest method (RF) was carried out in randomForest [103]; the mean decrease accuracy (MDA) was used for assignment of feature relation to class difference. (Orthogonal) Partial Least Squares ((O)PLS-DA) was performed with ropls. Factor loadings (*p*) and variable importance in projection (VIP), used as statistics to access the relationship between features and parameters, were modeled [104]. For metabolite set enrichment analysis (MSEA), the fgsea algorithm was used [38].

Metabolite sets for metabolite pathways for *M. truncatula* were downloaded in the KEGG database [105] using *KEGGREST* [106]. List of metabolites for pathways were manually corrected: poorly represented or extra-large sets were excluded; for some metabolites, obligatory needful pathways were added, and compounds identified up to class (hexose, disaccharide, among others) were joined relevant pathways (Tables S1 and S2).

Graphs were built using the Cytoscape software [107].

## 5. Conclusions

The results of the study indicate the suitability of using the highly sensitive *M. lupulina* MlS-1 line as a host plant to investigate the mechanisms of arbuscular mycorrhiza formation. Apparently, the high responsiveness of *M. lupulina* allowed us to demonstrate not only a mycorrhiza-induced intensification of development but also an acceleration of the change in phenophases. Along with the strong phenotypic expression, the intensification of biophysical (photosynthesis indicators) and biochemical processes (accumulation of pigments, etc.) is shown. It should be emphasized that the estimated mycorrhization efficiency is a dynamically changing parameter which, for most indicators, grows at the stooling stage and reaches a maximum at the SBI at 38 DAS. The use of GS-MS detection allowed not only for characterization of the metabolome of *M. lupulina* leaves but also to provide detailed metabolic profiling. The obtained data strongly indicate the presence of dynamic biochemical rearrangements during plant development (with a change of phenophases) and with arbuscular mycorrhiza development. It was concluded that the metabolic changes realized during the development of the host plant are more extensive than those caused by the emerging arbuscular mycorrhiza, even under the condition of high responsiveness on the part of the plant under analysis. Most of the biochemical processes predicted by the KEGG database are realized synchronously during AM formation and in its absence. Presumably, they mediate the possibility of mycorrhizal *M. lupulina* plants passing the transition to the next phenophase at a relatively “younger metabolic age”. The application of complex mathematical analysis made it possible to identify the clustering of various groups of metabolites and demonstrate the central importance of the carbohydrate cluster.

## Data Availability

Not applicable.

## Supplementary Materials

The following are available online at https://www.mdpi.com/article/10.3390/plants10112506/s1, Figure S1. The height of the main stem (B) and the number of internodes (D) per one *M. lupulina* plant. “−AM” is the variant without AM fungus inoculation, “+AM” is the variant inoculated with *R. irregularis* AM fungus. The axis of the abscissa is DAS. Figure S2. Symbiotic AM-efficiency calculated as the increase in fresh weight (E_FW_) of aerial parts, the increase in plant height (E_PH_), the increase in the number of leaves (E_NL_), the increase in the number of internodes (E_NI_), the increase in the area of leaves (E_AL_) per 1 *M. lupulina* MlS-1 plant. The axis of the abscissa is DAS. Figure S3. The effective photochemical quantum yield of photosystem II (*Y(II)* = *Fv’*/*Fm’*); (A) and area of leaves (B). The axis of the abscissa is DAS, see other notes in Figure 1. Figure S4. The chlorophyll *a* (*Chl-a*), chlorophyll *b* (*Chl-b*) and total carotenoid content (*Σ**Car*) in *M. lupulina* leaf tissues. *—significant (*p* < 0.05) differences in the average values of pigment content in “−AM” variant (without AM) and “+AM” variant (inoculated with *R. irregularis*). The axis of the abscissa is DAS. Figure S5. The ratio of the total carotenoid content (*Σ**Car*) to the total chlorophyll content(*Σ**Chla+b*). *—significant (*p* < 0.05) differences in the average values of pigment content in “−AM” variant (without AM) and “+AM” variant (inoculated with *R. irregularis*). The axis of the abscissa is DAS. Figure S6. Representation of metabolite profiles of leaves sampled in mycorrhized (+AM) and nonmycorrhized (−AM) *Medicago lupulina* plants in low-dimensional spaces. PCA—score plots, %—percent of variance. MDS—metabolite profiles in the space were revealed using multidimensional scaling (MDS) with 1-*r* as a measure of distance between observations, where *r* is Pearson’s correlation coefficient. Ellipses—90% CI. Figure S7. Comparative analysis of the metabolom dynamics in the mycorrhized (+AM) and not mycorrhizied (-AM) plants. SUS-plot of the loadings from OPLS models (with time as response) for −AM and +AM. Figure S8. Enrichment analysis based on OPLS-DA loadings *p*. Up arrows refer to positive NES that means generally, upregulations under mycorrhization or at later point, pathways clustered by a number of common metabolites in profile as proximity measure. Figure S9. Comparing metabolomic shifts between different phases. SUS-plot of loadings from OPLS-DA for corresponding pairs of phases: 1st leaf (1L)—3rd leaf initiation (3LI), 3rd leaf initiation (3LI)—stooling initaitation (SI), stooling initaitation (SI)—stem branching initiation (SBI), stooling branching initiation (SBI)—flowering inititiation (FI). Positive loadings correspond to higher level at later stage. Figure S10. Comparison of mycorrhization effects at different phases: stooling initiation (SI)/1st leaf (1L) (A); SI/flowering initiation (FI) (B). SUS-plot of loadings from OPLS-DA for +AM and −AM. Positive loadings correspond to higher level at +AM. Figure S11. Comparative analysis of metabolomic shifts between common phases: 1st leaf (1L)—stooling initiation (SI) (A), SI—flowering initiation (FI) of −AM (control) and +AM plants (B). SUS-plot of loadings from OPLS-DA for corresponding phase pairs (see SUS titles). Positive loadings correspond to higher level at a later stage. Figure S12. Comparative analysis of AM effect and developmental metabolic alterations. SUS-plot of the loadings from OPLS-DA models for comparison of −AM and +AM plants at SI (absciss) and for comparison of control plants at 3LI and SI. Figure S13. Number strong (|r| > 0.7) correlations for each metabolite under +AM and −AM development. Metabolites with high rates of difference are marked. Figure S14. Steps of fungal inoculum preparation. Table S1. Metabolite content normilized per saple median. Table S2. List of metabolic pathways and compounds identified up to class. Table S3. Agrochemical soil characteristics.
