# Supplementary material for: Mycorrhiza-Induced Alterations in Metabolome of Medicago lupulina Leaves during Symbiosis Development
Source: Plants (Basel). 2021 Nov 18;10(11):2506. doi: 10.3390/plants10112506 (PMC8617643; doi:10.3390/plants10112506)
Supplement: Supplementary file 1 [file plants-10-02506-s001.zip › plants-1403513-supplementary.pdf]

Supplementary Materials:

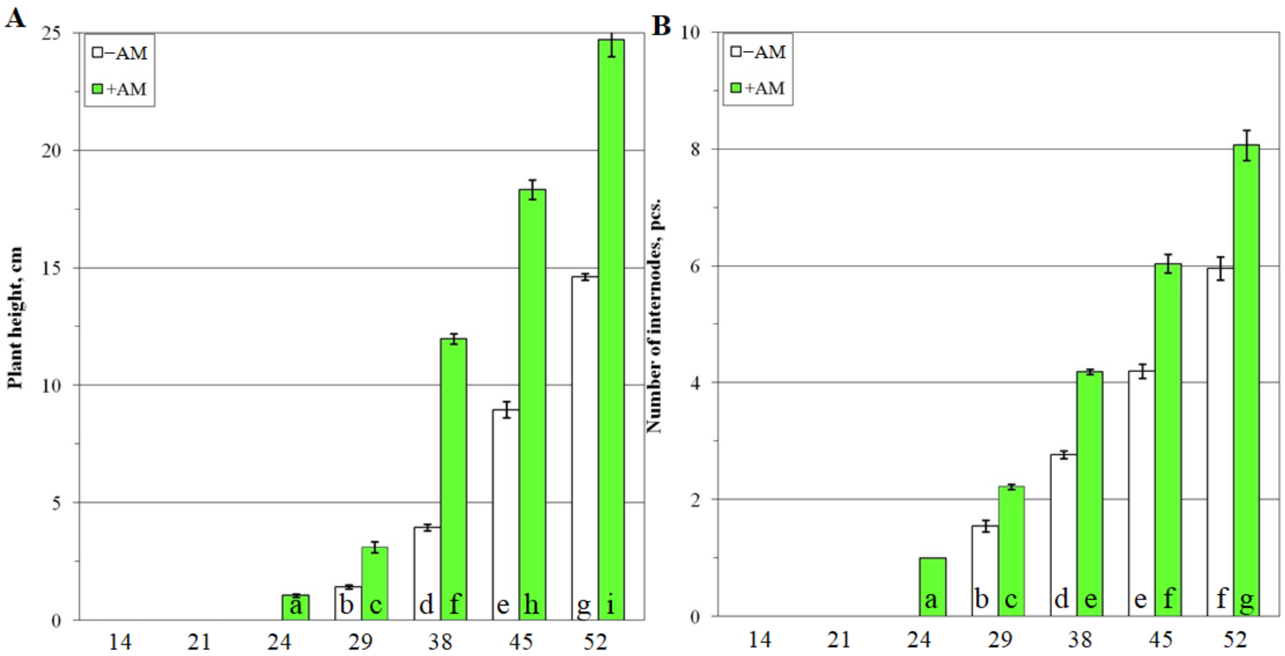

Figure S1. The height of the main stem (B) and the number of internodes (D) per one *M. lupulina* plant. "–AM" is the variant without AM fungus inoculation, "+AM" is the variant inoculated with *R. irregularis* AM fungus. The axis of the abscissa is DAS.

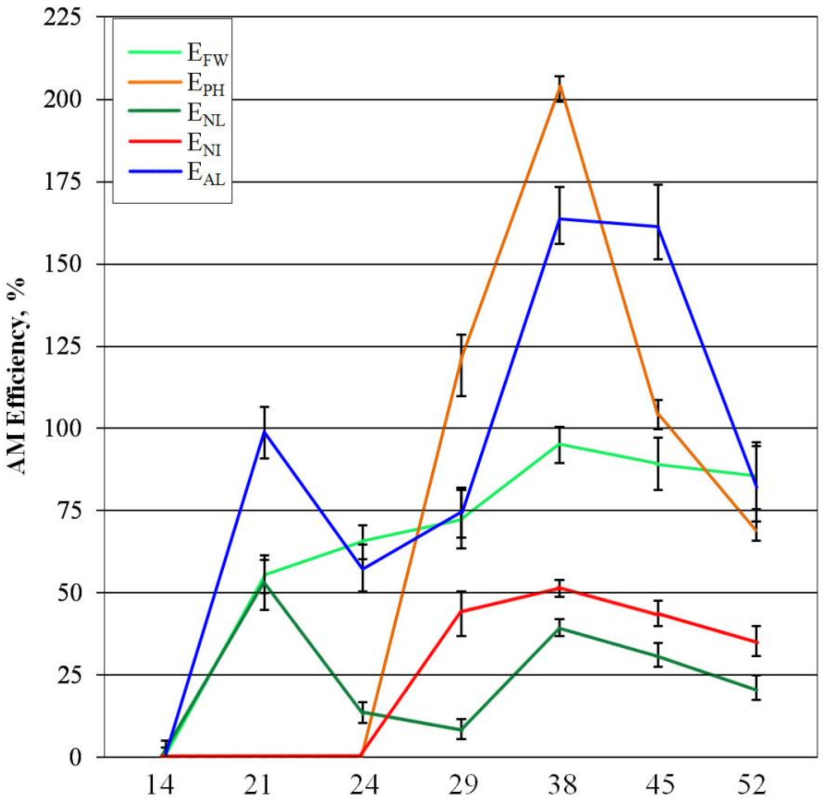

Figure S2. Symbiotic AM-efficiency calculated as the increase in fresh weight (E_{FW}) of aerial parts, the increase in plant height (E_{PH}), the increase in the number of leaves (E_{NL}), the increase in the number of internodes (E_{NI}), the increase in the area of leaves (E_{AL}) per 1 *M. lupulina* MlS-1 plant. The axis of the abscissa is DAS.

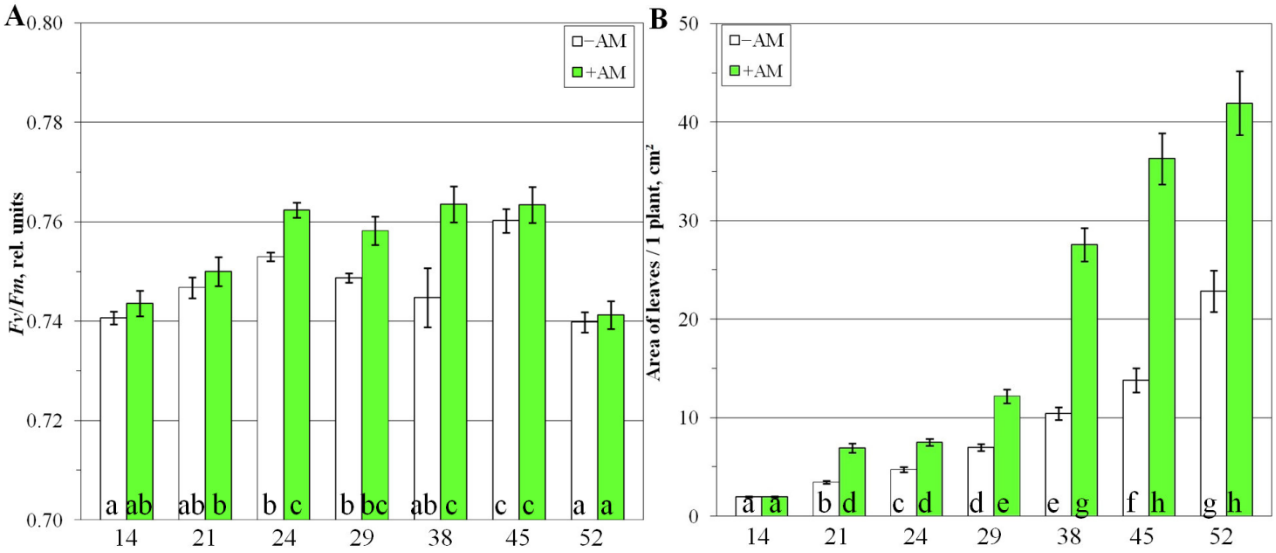

Figure S3. The effective photochemical quantum yield of photosystem II (*Y(II)* = *Fv′*/*Fm′*; **A**) and area of leaves (**B**). The axis of the abscissa is DAS, see other notes in Fig. 1.

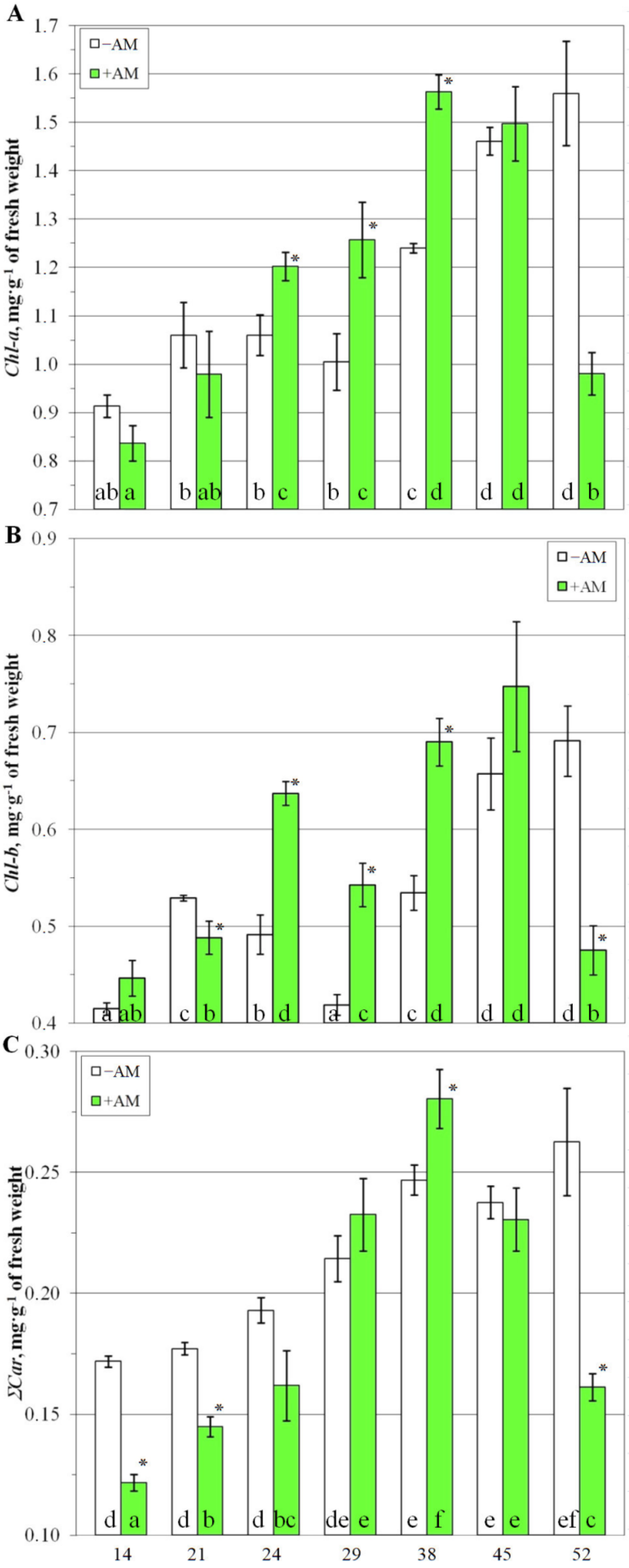

Figure S4. The chlorophyll *a* (*Chl-a*), chlorophyll *b* (*Chl-b*) and total carotenoid content (Σ*Car*) in *M. lupulina* leaf tissues. *—significant ($p < 0.05$) differences in the average values of pigment content in "–AM" variant (without AM) and "+AM" variant (inoculated with *R. irregularis*). The axis of the abscissa is DAS.

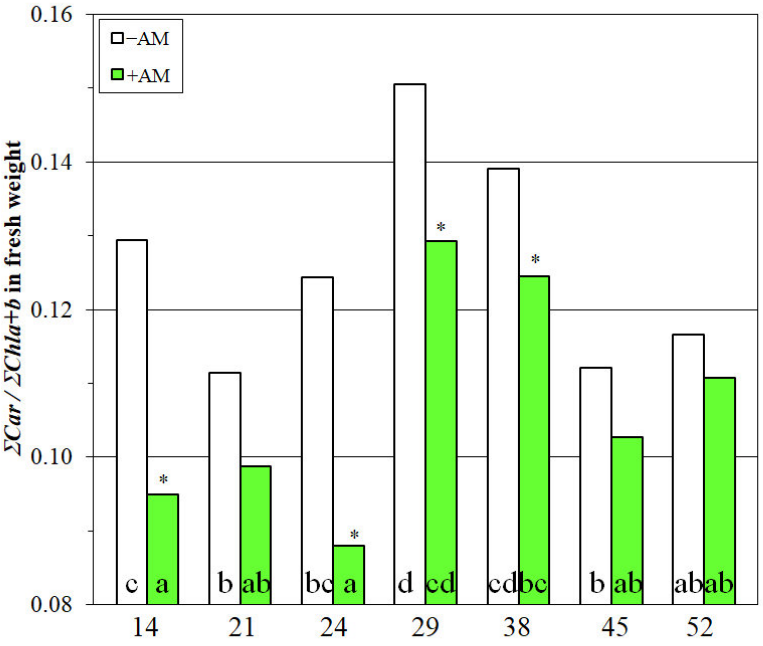

Figure S5. The ratio of the total carotenoid content (Σ*Car*) to the total chlorophyll content(Σ*Chla*+*b*). *—significant ($p < 0.05$) differences in the average values of pigment content in "−AM" variant (without AM) and "+AM" variant (inoculated with *R. irregularis*). The axis of the abscissa is DAS.

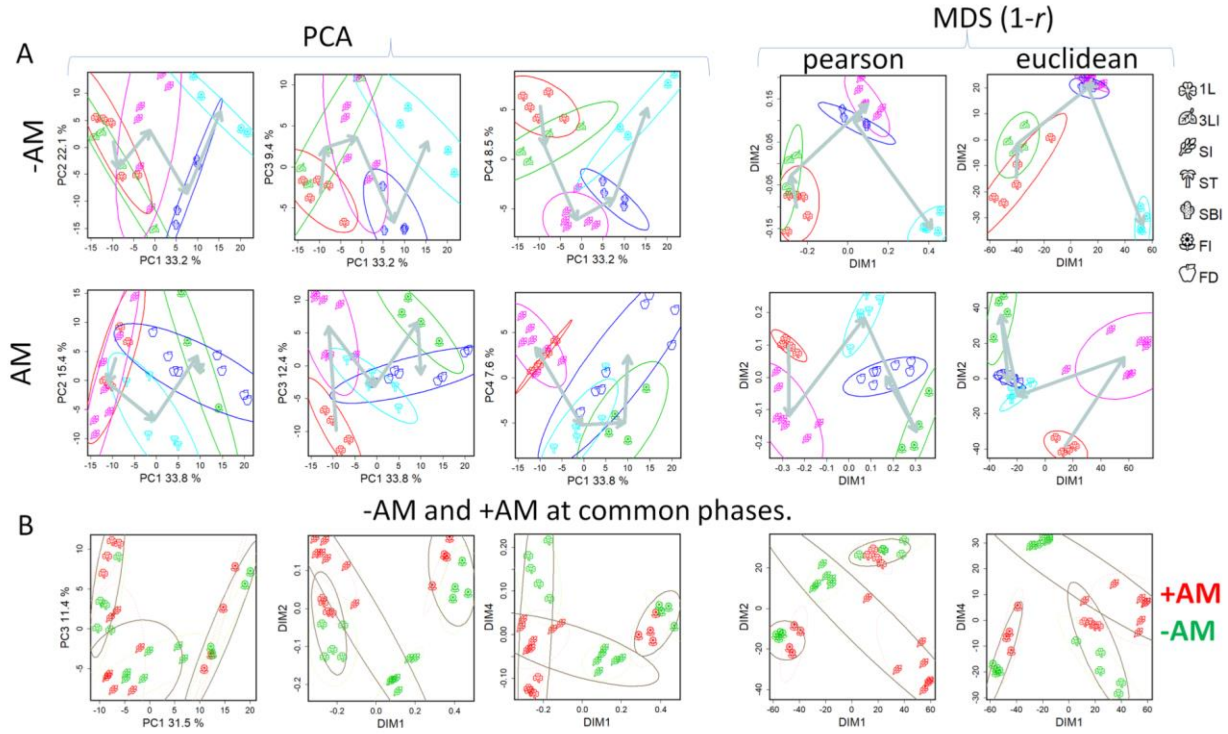

Figure S6. Representation of metabolite profiles of leaves sampled in mycorrhized (+AM) and nonmycorrhized (−AM) *Medicago lupulina* plants in low-dimensional spaces. PCA—score plots, %—percent of variance. MDS—metabolite profiles in the space were revealed using multidimensional scaling (MDS) with 1-*r* as a measure of distance between observations, where *r* is Pearson's correlation coefficient. Ellipses—90% CI.

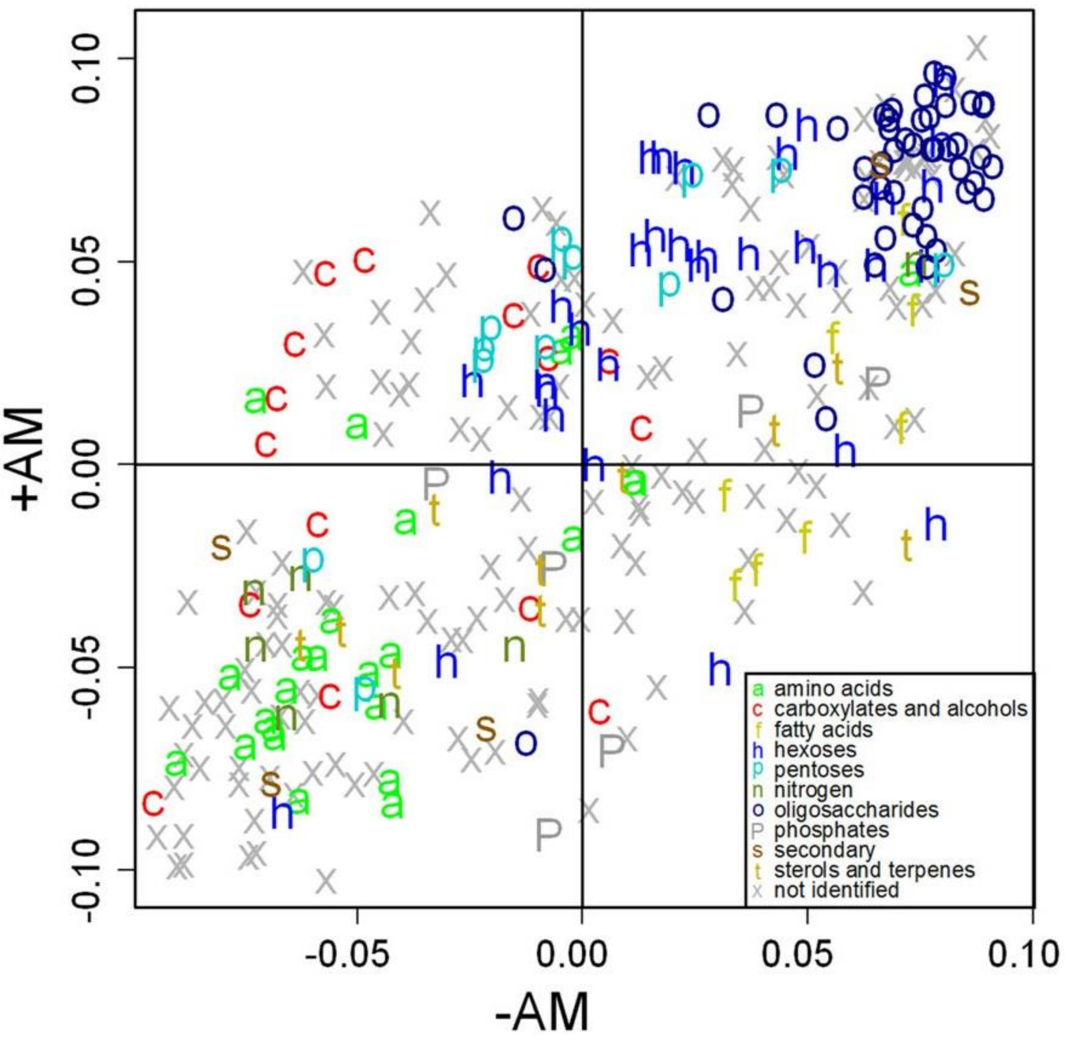

Figure S7. Comparative analysis of the metabolom dynamics in the mycorrhized (+AM) and not mycorrhizied (−AM) plants. SUS-plot of the loadings from OPLS models (with time as response) for −AM and +AM.

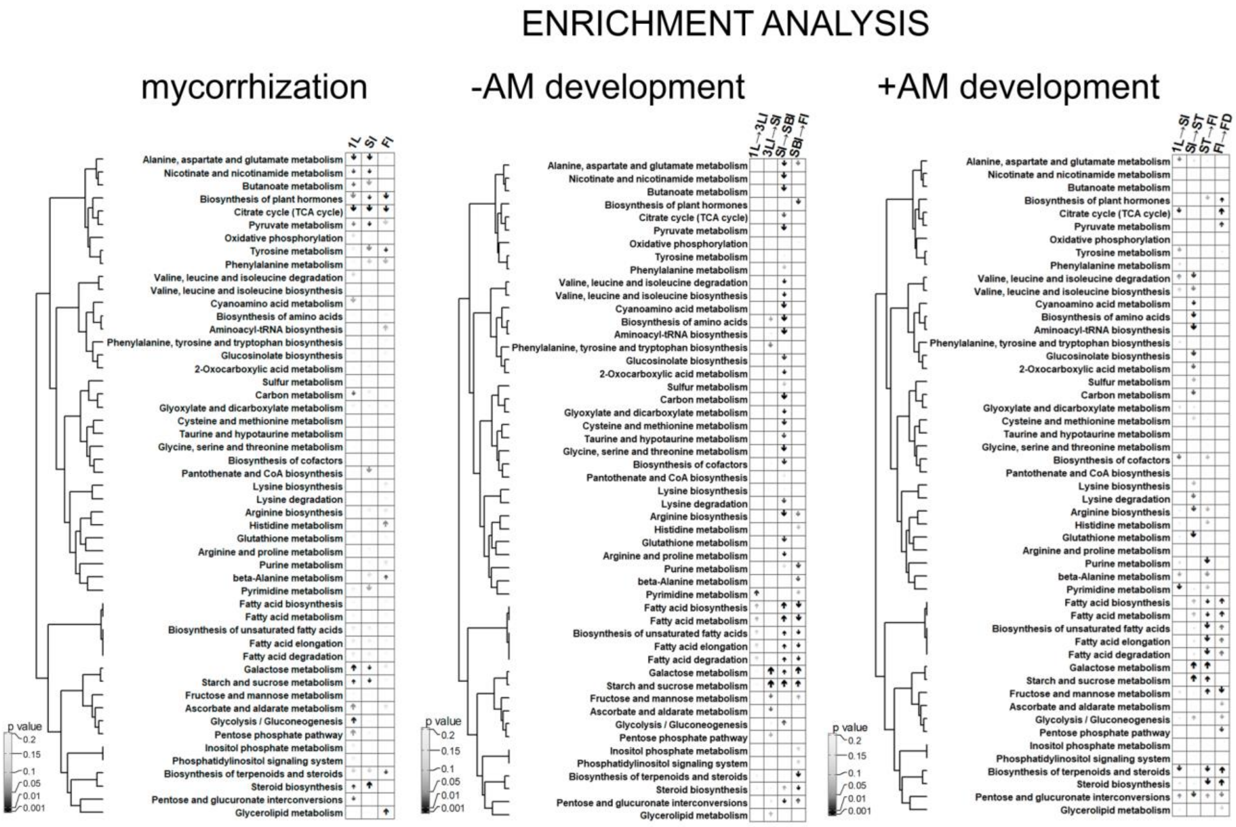

Figure S8. Enrichment analysis based on OPLS-DA loadings *p*. Up arrows refer to positive NES that means generally, upregulations under mycorrhization or at later point, pathways clustered by a number of common metabolites in profile as proximity measure.

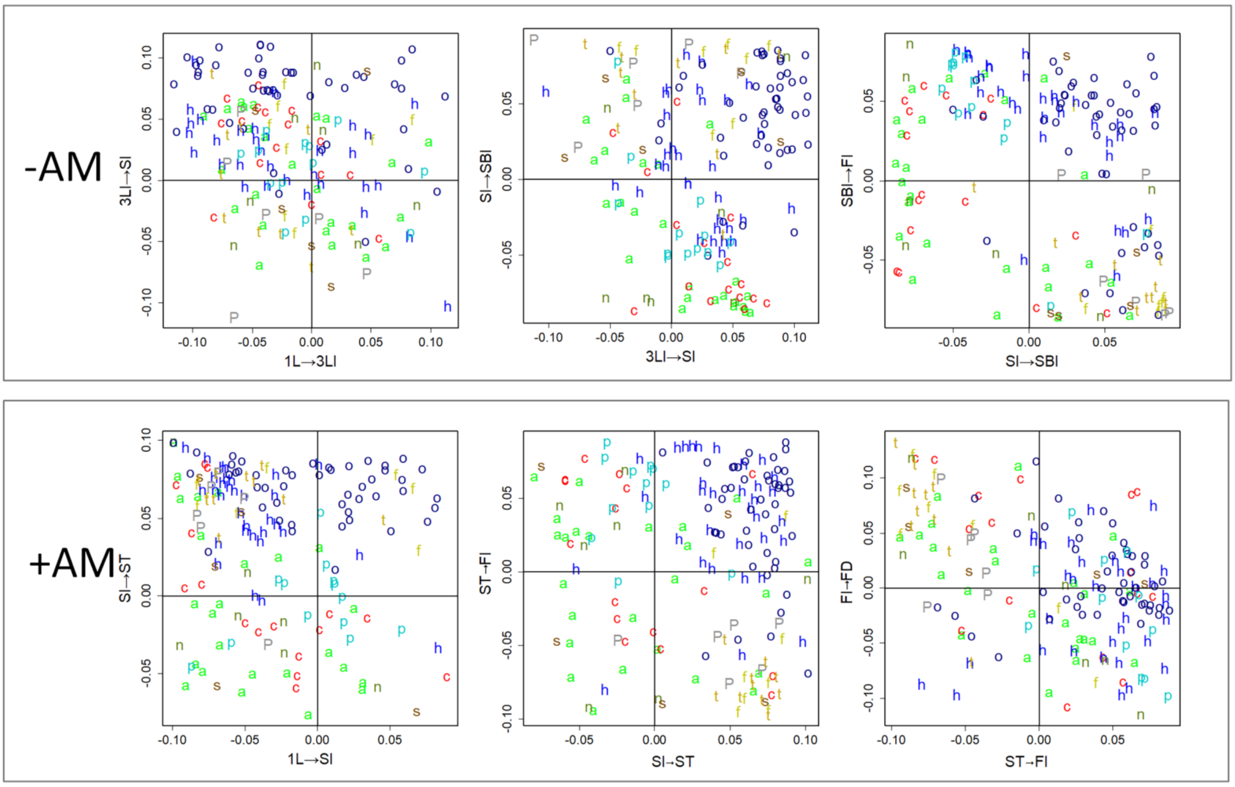

Figure S9. Comparing metabolomic shifts between different phases. SUS-plot of loadings from OPLS-DA for corresponding pairs of phases: 1st leaf (1L)—3rd leaf initiation (3LI), 3rd leaf initiation (3LI)—stooling initaitation (SI), stooling initaitation (SI)—stem branching initiation (SBI), stooling branching initiation (SBI)—flowering inititiation (FI). Positive loadings correspond to higher level at later stage.

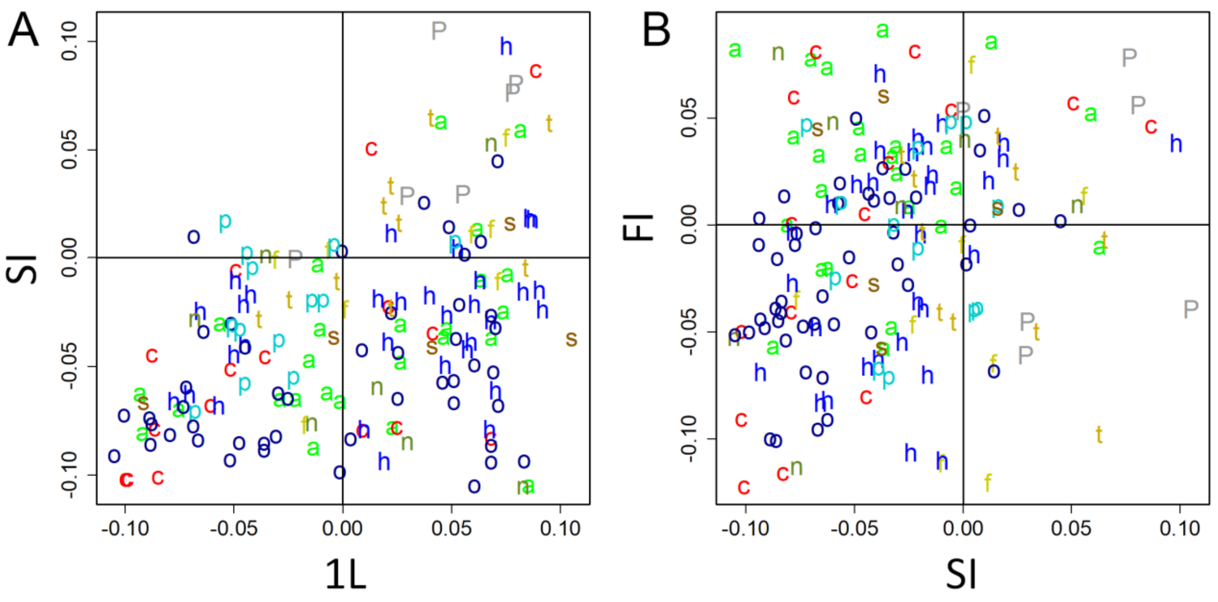

Figure S10. Comparison of mycorrhization effects at different phases: stooling initiation (SI) /1st leaf (1L) (**A**); SI / flowering initiation (FI) (**B**). SUS-plot of loadings from OPLS-DA for +AM and –AM. Positive loadings correspond to higher level at +AM.

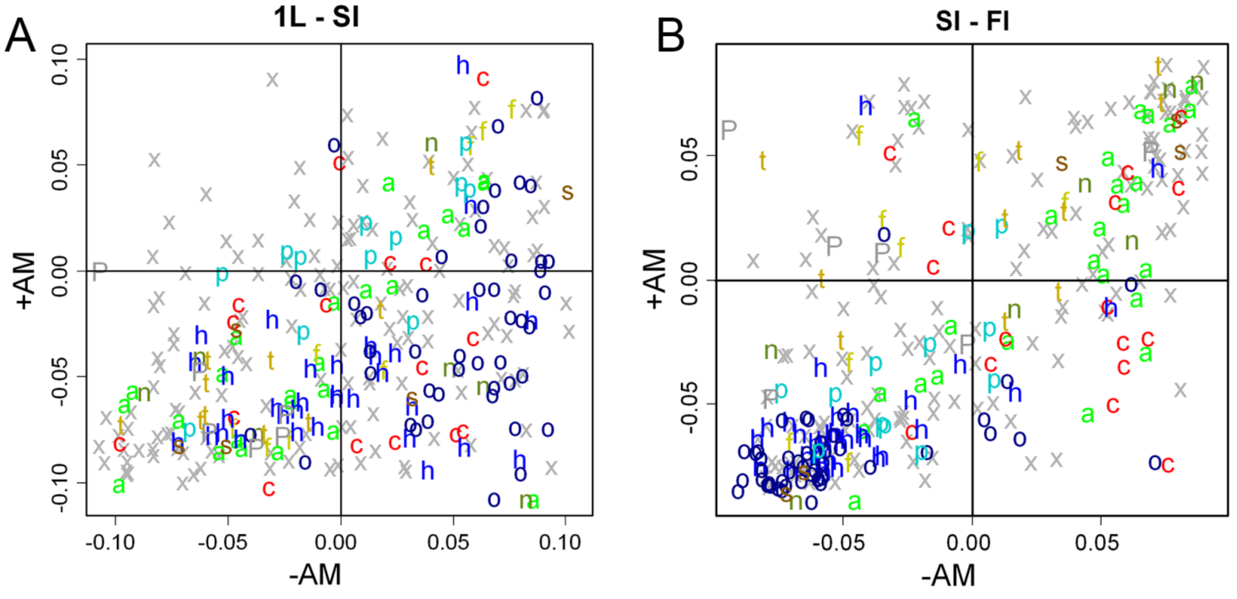

Figure S11. Comparative analysis of metabolomic shifts between common phases: 1st leaf (1L)—stooling initiation (SI) (**A**), SI—flowering initiation (FI) of –AM (control) and +AM plants (**B**). SUS-plot of loadings from OPLS-DA for corresponding phase pairs (see SUS titles). Positive loadings correspond to higher level at a later stage.

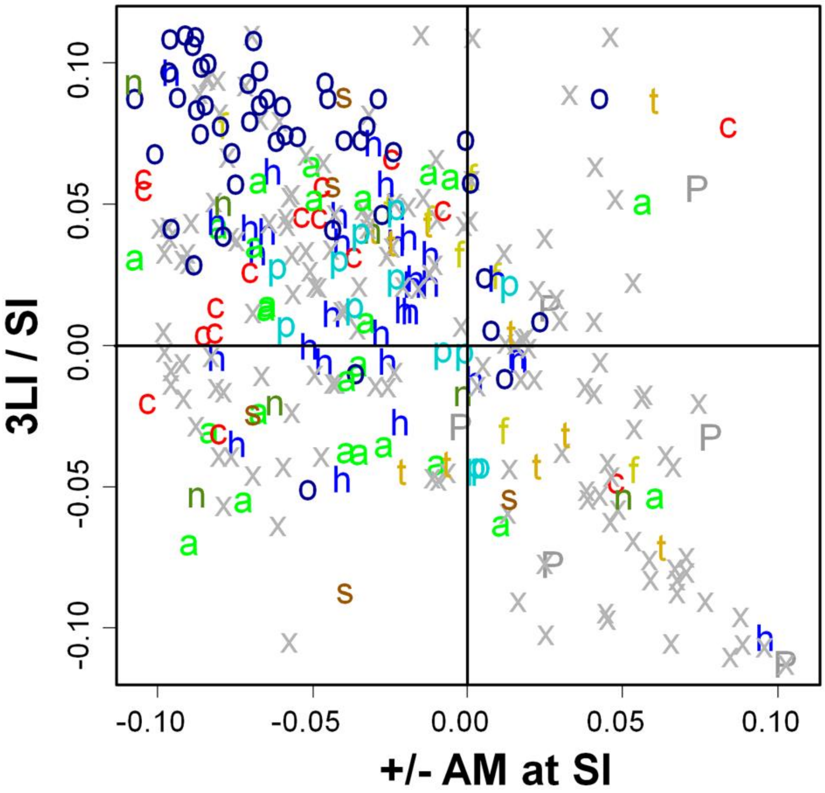

Figure S12. Comparative analysis of AM effect and developmental metabolic alterations. SUS-plot of the loadings from OPLS-DA models for comparison of −AM and +AM plants at SI (absciss) and for comparison of control plants at 3LI and SI.

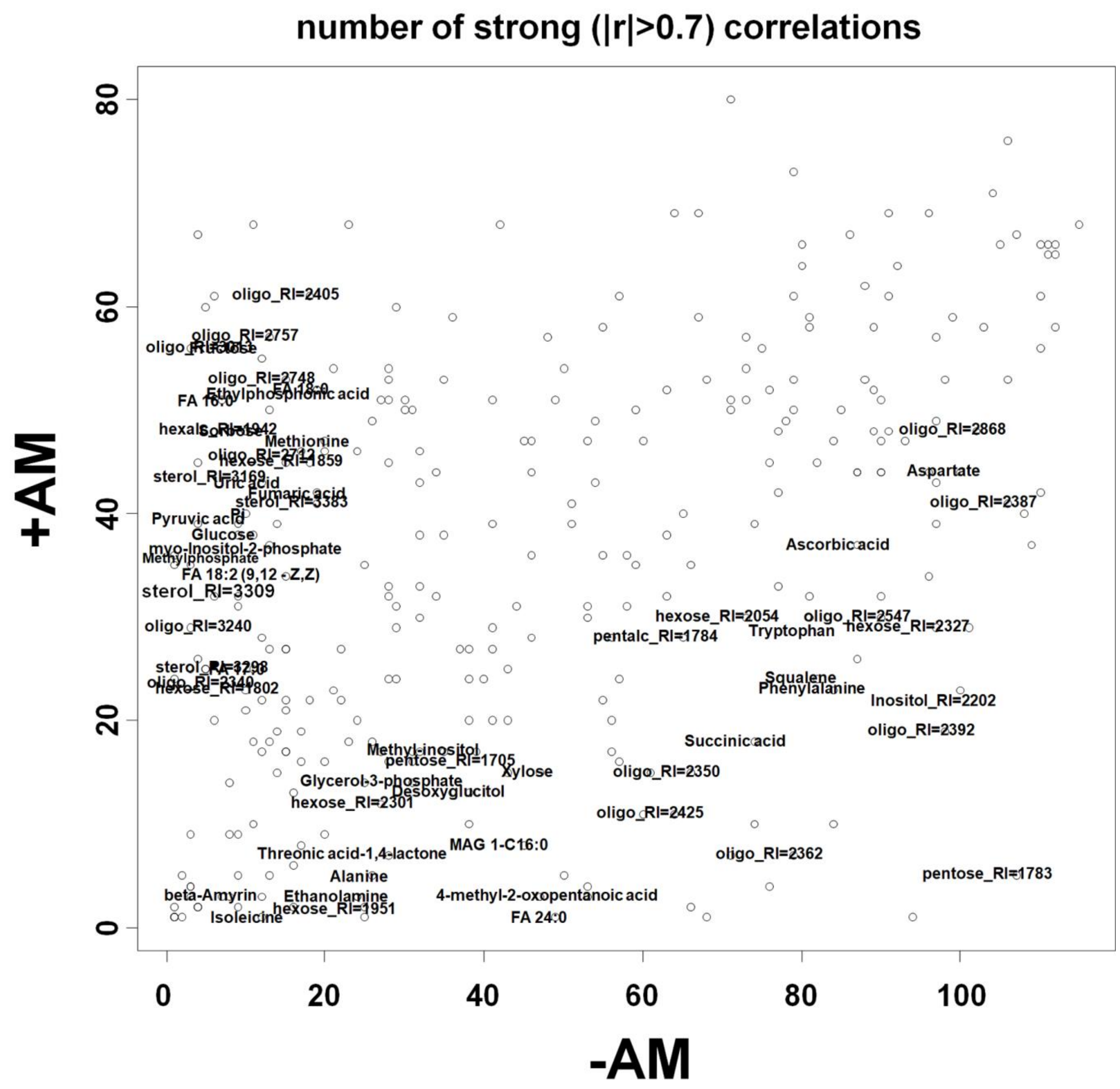

Figure S13. Number strong (|r| > 0.7) correlations for each metabolite under +AM and –AM development. Metabolites with high rates of difference are marked.

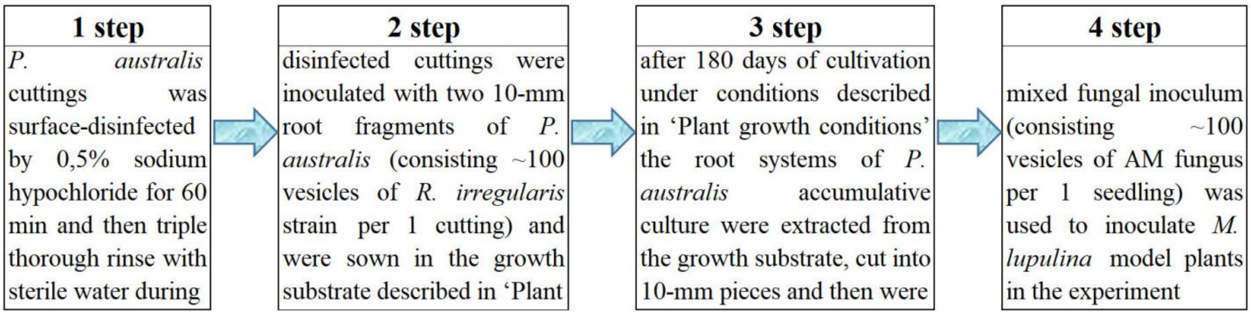

Figure S14. Steps of fungal inoculum preparation.

Table S3. Agrochemical soil characteristics.

Soil type	P_2O_5 content, mg/kg	K_2O content,	Organic matter content,	pH_{KCl}	pH_{H2O}

			mg/kg	%		
**Sod-podzolic loam-poor**	23	(very low)	78	3.64	6.44	7.28
